# Functional annotation and distribution overview of RNA families in 27 *Streptococcus agalactiae* genomes

**DOI:** 10.1186/s12864-018-4951-z

**Published:** 2018-07-28

**Authors:** Ivan Rodrigo Wolf, Alexandre Rossi Paschoal, Cecilia Quiroga, Douglas Silva Domingues, Rogério Fernandes de Souza, Lucienne Garcia Pretto-Giordano, Laurival Antonio Vilas-Boas

**Affiliations:** 1 Departamento de Biologia Geral, Centro de Ciências Biológicas, Universidade Estadual de Londrina, Londrina, Paraná Brazil; 2 Universidade Tecnológica Federal do Paraná, Campus Cornélio Procópio, Cornélio Procópio, Paraná, Brazil; 3 Universidad de Buenos Aires, Consejo Nacional de Investigaciones Científicas y Tecnológicas, Instituto de Investigaciones en Microbiología y Parasitología Médica (IMPAM), Facultad de Medicina, Buenos Aires, Argentina; 4 Departamento de Botânica, Instituto de Biociências de Rio Claro, Universidade Estadual Paulista Júlio de Mesquita Filho, Rio Claro, São Paulo Brazil; 5 Departamento de Medicina Veterinária Preventiva, Universidade Estadual de Londrina, Londrina, Paraná Brazil

**Keywords:** *Streptococcus*, Non-coding RNAs, Pan-genome, RNA families, Transcriptome

## Abstract

**Background:**

*Streptococcus agalactiae,* also known *as* Group B *Streptococcus* (GBS), is a Gram-positive bacterium that colonizes the gastrointestinal and genitourinary tract of humans. This bacterium has also been isolated from various animals, such as fish and cattle. Non-coding RNAs (ncRNAs) can act as regulators of gene expression in bacteria, such as *Streptococcus pneumonia*e and *Streptococcus pyogenes.* However, little is known about the genomic distribution of ncRNAs and RNA families in *S. agalactiae*.

**Results:**

Comparative genome analysis of 27 *S. agalactiae* strains showed more than 5 thousand genomic regions identified and classified as Core, Exclusive, and Shared genome sequences. We identified 27 to 89 RNA families per genome distributed over these regions, from these, 25 were in Core regions while Shared and Exclusive regions showed variations amongst strains. We propose that the amount and type of ncRNA present in each genome can provide a pattern to contribute in the identification of the clonal types.

**Conclusions:**

The identification of RNA families provides an insight over ncRNAs, sRNAs and ribozymes function, that can be further explored as targets for antibiotic development or studied in gene regulation of cellular processes. RNA families could be considered as markers to determine infection capabilities of different strains. Lastly, pan-genome analysis of GBS including the full range of functional transcripts provides a broader approach in the understanding of this pathogen.

**Electronic supplementary material:**

The online version of this article (10.1186/s12864-018-4951-z) contains supplementary material, which is available to authorized users.

## Background

*Streptococcus agalactiae* (GBS) is a Gram-positive bacterium that can colonize the gastrointestinal and urogenital tracts of humans. Despite this natural association, *S. agalactiae* infections are common in neonates [1], immunosuppressed adults and the elderly [2].

GBS infections are not restricted to humans. Historically, this microorganism is a causative agent of bovine mastitis and may infect several fish species, causing production losses in aquaculture systems [3–5], besides affecting other animals such as mice, dogs, cats and horses (as seen in [6, 7]).

This diversity of environments in which *S. agalactiae* can be found depends on its ability to control regulatory networks responsible for avoiding the host’s immune system and to acquire nutrients [8].

Transcriptome *microarray* analysis revealed that *S. agalactiae* strains were able to regulate their transcriptional levels according to different factors, such as growth medium temperature [9], the presence of human blood [10] and the presence of human amniotic fluid [11]. On the other hand, in cultures with high glucose concentration and absence of host’s stimuli, genes related to virulence and stress response were down regulated [8].

The development of new techniques, such as *tiling array* and ribosomal RNA depletion, has made it possible to verify a great diversity of non-translated RNAs in bacteria [12]. The number and diversity of these elements led to the organization of these RNAs in families (Rfams) that increased from 25 to over 2200 in 10 years [13].

Research studies have shown that non-coding RNAs (ncRNAs) can modulate gene expression in bacteria, as well as coordinate adaptive processes that responds to environmental changes and control target gene expression [14, 15]. Consequently, ncRNAs play key roles in gene regulatory networks that responds to environmental stimuli, and this occurs in several pathogenic bacteria, such as *Vibrio cholerae, Salmonella* Typhimurium*, Staphylococcus aureus,* and various species of *Streptococcus* [16–19].

At the time of this writing, 18 global analyses aiming to investigate whether non-coding RNAs are available in *Streptococcus* have been accomplished [19, 20]. The number of ncRNAs detected in these studies range from 10 to 900 [19]. However, more than half of these studies focus on *Streptococcus pneumoniae* and *S. pyogenes* (as seen in [19]). On the other hand, only two reports are currently available for a single strain of *S. agalactiae*. In the first one a model developed by the authors found 197 candidates [21], and in the second one differential RNA-Seq data (dRNA-Seq) revealed more than 100 ncRNAs [22].

Here we report the first comparative genome analysis of ncRNA of *S. agalactiae*. The data showed a compact Core genome region interleaved with Shared and Exclusive regions. Our predictions showed 27 to 89 Rfams per genome where 25 are always present in Core genome regions. Some of the RNA families identified were related to mobile genetic elements and essential functions like iron homeostasis, sugar metabolism and virulence genes regulation. Moreover 4 RNA families are reported for the first time in the Core region of strain NEM316.

## Methods

### ncRNAs selection

In this study RNA families (Rfam) refer to families of untranslated RNAs from Rfam database [23]; among the Rfam those that have 500pb or less were considered small RNAs (sRNAs).

### Genomic sequences

This study comprised 27 genomes falling either into the “Complete Genome” or “Complete Chromosome” level available from the National Center for Biotechnology Information (NCBI) database [24] on January 24, 2016. Information on the isolation source of each genome was obtained from the same database and simplified in Table 1.

**Table 1:** Number of RNA families detected in each genome region and each strain, and total annotations with transcriptional signals

Strain^a^	Accession^b^	E	S	Tnc	Cluster^c^	MLST^d^	Source^e^
GD201008–001	CP003810	0	36	61	cluster2	7	Fish
GBS6	CP007572	6	54	85	cluster2	22	Mammal/clinical
GBS2-NM	CP007571	3	46	74	cluster2	22	Mammal/clinical
GBS1-NY	CP007570	10	54	89	cluster2	22	Mammal/clinical
HN016	CP011325	0	36	61	cluster2	10	Fish
YM001	CP011326	2	34	61	cluster2	10	Fish
GX064	CP011327	3	29	57	cluster2	10	Fish
2603 V/R	AE009948	0	10	35	cluster1	110	Mammal/clinical
A909	CP000114	1	7	33	cluster1	7	Mammal/clinical
NEM316	AL732656	2	5	32	cluster1	23	Mammal/clinical
09mas018883	HF952104	1	6	32	cluster1	1	Mammal/cattle
ILRI112	HF952106	4	3	32	cluster1	617	Mammal/camels
ILRI005	HF952105	3	3	31	cluster1	609	Mammal/camels
COH1	HG939456	0	13	38	cluster1	110	Mammal/clinical
NGBS061	CP007631	1	8	34	cluster1	459	Mammal/clinical
NGBS572	CP007632	0	13	38	cluster1	452	Mammal/clinical
CNCTC_10/84	CP006910	2	2	29	cluster1	26	Mammal/clinical
SS1	CP010867	0	6	31	cluster1	1	Mammal/clinical
H002	CP011329	0	7	32	cluster1	736	Mammal/clinical
GBS85147	CP010319	3	4	32	cluster1	103	Mammal/clinical
SG-M1	CP012419	1	4	30	cluster1	283	Mammal/clinical
GBS_ST-1	CP013202	0	8	33	cluster1	1	Mammal/canine
SA20–06	CP003919	0	2	27	cluster0	553	Fish
138P	CP007482	0	2	27	cluster0	261	Fish
138spar	CP007565	0	2	27	cluster0	261	Fish
2–22	FO393392	0	2	27	cluster0	261	Fish
GX026	CP011328	0	2	27	cluster0	261	Fish

E: Total RNA families detected in Exclusive Regions; S: Total RNA families detected in Shared regions; Tnc: Total RNA families detected in specific strain

^a^Name of strain deposited in the NCBI database

^b^NCBI accession number

^c^Grouping according to *cluster* analysis performed in Weka

^d^Multilocus sequence typing information for each strain

^e^Strain source

Multilocus sequence typing (MLST) data was retrieved from PubMLST (https://pubmlst.org/bigsdb?db=pubmlst_sagalactiae_seqdef&page=sequenceQuery) [25] (for MLST information see Additional file 1).

### Determination of Core, exclusive and shared genomic regions

Mauve [26] software was used to align the 27 *S. agalactiae* genomes and the strain NEM316 was used as reference genome for alignments since its genome has been completely sequenced [27], there is transcriptomic data available [22, 28] and was taken as a model for the search of non-coding RNAs in this group [21].

Genomic coordinates were extracted from Mauve output (backbone file) and classified into three kinds of sequences (I) Core: regions present in all genomes; (II) Exclusive: regions occurring only in one genome; (III) Shared: regions present in two or more, but not in all genomes. To avoid misclassification of small regions that happen in the normal evolution of strains, such as single nucleotide polymorphisms (SNPs), only regions larger than 10 base pairs (bp) were extracted. To visually inspect the distribution of both genomic and ncRNA regions, annotations were plotted with the software Circos [29].

### Horizontal gene transfer analysis

To investigate the existence of probable horizontally transferred regions into the Exclusive regions of each genome, we used the Alien Hunter [30, 31], PHASTER [32] and IslandViewer [33]. Alien Hunter regions with the output tag “probably overlapping rRNA operon” were disregarded.

### Annotation of Rfams

RNA families identification and annotation was performed with Infernal v1.1 [34] with default settings, and the Rfam database v12.0 [23]. Rfams were restricted to 147 families already identified in the order Lactobacillales (Additional file 2), as recommended by Nawrocki in [35]. The family with the lowest e-value was selected in case of overlap [35]. Also, the nocoRNAc [36] was used with default settings to predict possible promoter and terminator regions around the reported Rfams. It should be noted that group II intron prediction uses several RNA families from Rfam database (group II D1-D4 1 to 7 and intron_gpII), thus estimations were then curated (Additional file 3).

Intergenic regions of all genomes were obtained with Artemis genome browser [37] to evaluate identified RNA families overlapping these regions.

The overlap between RNA families found for strain NEM316 were compared to the data provided by Pichon et al. [21] and Rosinski-Chupin et al. [22]. The resulting RNA families for NEM316 were tagged in the annotation files (Additional file 4) indicating in which study they were observed.

Annotated RNA family’s coordinates were compared to Core, Exclusive and Shared regions; when the RNA family coordinates overlap the genome region, the count for that region was increased by one.

To investigate the relationship between the number of RNA families and genome sizes, correlation analysis was performed after Shapiro-Wilk test of normality over the variables of total number of RNA families and genome size (Table 1) in R statistical environment [38] with package Hmisc.

### Transcription evidence

We obtained the RNA-Seq data for strain NEM316 from NCBI Sequence Read Archive (SRA) (Accession Numbers: SRX315261, SRX315262, SRX315263, SRX315264 and SRX315265) to verify if the RNA families regions were capable of being transcribed to exert their functional role. Sequences were cleaned with Trimmoatic [39] and mapped on NEM316 strain genome with Bowtie2 [40] using the “--very-sensitive” flag. The coverage per base was obtained with SAMtools [41]. The raw number of reads over RNA family’s annotation extension was plotted for visual inspection of transcriptional activity. The coverage was also plotted for two ranges, of 800 base pairs (bp) and 1600 bp, surrounding the RNA family annotation using the “*stat = “coverage”*” argument for the *autoplot* function of ggbio package of Bioconductor [42]. If there was read coverage, i.e. reads mapped over the region with a RNA family annotated, the RNA family was considered real due to transcription activity on site.

### Cluster analysis

The expectation-maximization (EM) algorithm was applied taking into account the genome size, number of Shared regions, number of Exclusive regions, number of RNA families in Exclusive Regions, number of RNA families in Shared regions and total RNA families number as input attributes (Table 1, other attributes in Additional file 5) for data clustering in Weka software [43] using the default settings.

## Results

In order to study the pangenome of GBS strains we did a comparative genome analysis. We included in this study 27 genomes. We did a clustering analysis based on genomic features (for details see Methods), which led to the identification of three groups: cluster2, which were related to fish and mammalian strains; cluster1, related exclusively to a mammalian source; and cluster0 related to fish samples only (Table 1, the graphics in Additional file 6 show the mix of groups according to each attribute analyzed).

**Fig. 1:**
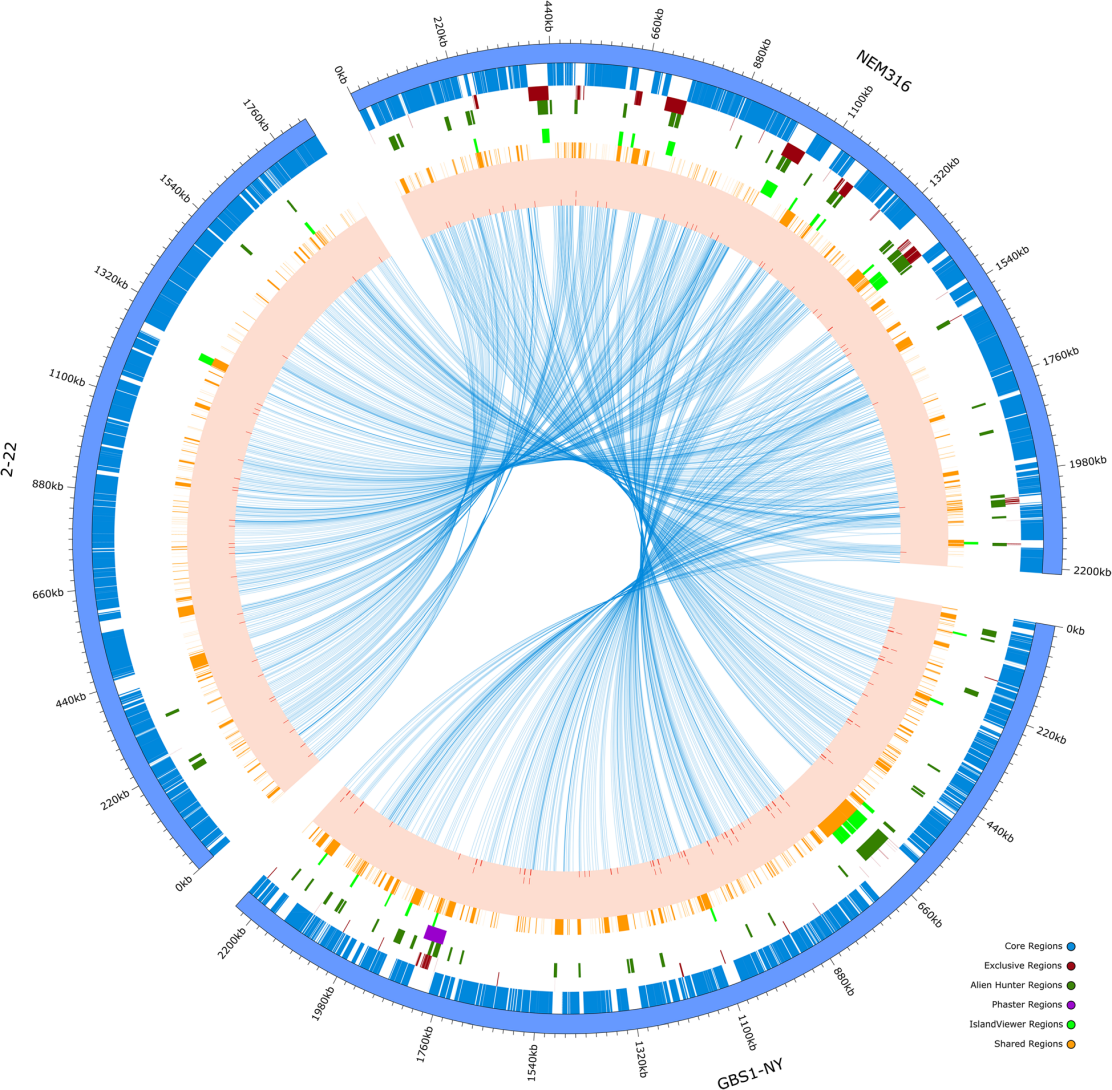
Circular visualization of three *S. agalactiae* genomes analyzed. The tracks, from outside to the inside of the circle, represent: (Track 1): chromosome extension; (Track 2): Core genome regions, present in all genomes analyzed; (Track 3): Exclusive regions, found only for that genome; (Tracks 4): probable horizontally transferred regions detected by Alien Hunter; (Tracks 5): probable horizontally transferred regions detected by Phaster; (Tracks 6): probable horizontally transferred regions detected by IslandViewer; (Track 7): Shared regions, present in two or more genomes, but not in all; (Track 8): Bars indicate the position of ncRNAs identified in this study; (Track 9): Internal links representing the connection between the Core genome of all genomes to the reference genome utilized in the Mauve program

The alignments of 27 genomes allowed the classification of 5224 regions (amongst Core, Exclusive and Shared regions) distributed in all the analyzed genomes. Of those, 458 were classified as Core, 997 as Exclusive, and 3769 as Shared (Fig. 1, tracks 2, 3 and 7; for all genomes pictures refer to Additional file 7). The size of Core regions ranged from 10 to 39,361 bp, while Shared and Exclusive regions ranges from 10 to 21,935 bp and 19 to 47,076 bp respectively. To avoid information overload, only one genome of each cluster (Table 1) was exhibited, the image with all genomes together and individual genome plots can be found in Additional file 7. Shared regions were found scattered throughout the genome. Moreover, it was possible to observe that amongst the regions identified in each cluster, several of them formed mosaic structures that intercalates with the Core genome (Fig. 1 and Additional file 7). This suggests that horizontal transfer events might have occurred that contributed with genome evolution. Thus, we used softwares Alien Hunter, PHASTER and IslandViewer to identify regions supposedly acquired by horizontal transfer events; these regions overlapped with one another and with Shared and Exclusive Regions (Fig. 1 tracks 4, 5 and 6).

Then, we looked for the different non-coding RNA families present in GBS pangenome and evaluated their distribution in each genome. The search for these elements resulted in the identification of 27 to 89 RNA families per genome (Table 1). When considered by region, the Core, Shared, and Exclusive groups presented a minimum/maximum of 25/25, 2/54 and 0/10, respectively. The Shapiro-Wilk test of normality show that genome size and the number of RNA families did not follow a normal distribution (W = 0.89404, *p*-value = 0.009798 < 0.05 and W = 0.73345, p-value = 1.174e-05 < 0.05 respectively) then Spearman’s rank was selected for correlation analysis. A strong correlation between the genome size and the number of RNA families was found (Rho = 0.61), and this relationship was significant (*p*-value = 7e-04 < 0.05).

We then conducted a more detailed analysis of the identified Rfams in order to obtain additional information on GBS genome evolution. We detected 36 Rfams that overlapped at least with one of the three regions, which includes large ncRNAs, such as GOLLD, group II introns and RNAseP (Table 2). We focused in the distribution of the sRNAs in GBS genomes. This revealed that 24 out of 27 families were highly conserved (> 96%) (Additional file 3 and Additional file 5). Four sRNAs were found less frequently in the genomes included in this study: rli38 was present in 70% of genomes, SSRC38 in 48% of them, and rli28 and RatA in 55% of them. Other Rfams were found solely in one or two genomes, i.e. RNAout in strain A909, SpF01 in strain GBS85147, SpF39 in strain NGBS061, and Spf41 in strains CNCTC_10/84 and GBS85147. All sRNAs were fully or partially overlapping intergenic regions, except one family, namely sau-50.

**Table 2:** RNA families found in Core, Exclusive and Shared regions of *S. agalactiae* genomes

Core	Exclusive	Shared
23S-methyl	GOLLD	
6S	group-II-D1D4–1	
Asd	group-II-D1D4–3	
Bacteria_small_SRP	group-II-D1D4–5	
FMN	group-II-D1D4–7	
Glycine	Intron_gpII	
L10_leader		PyrR
L13_leader	RatA	
L17DE	rli28	
L20_leader	rli38	
L21_leader		SSRC38
Lacto-rpoB		
PreQ1		yjdF
preQ1-II	RNA-OUT	
Purine	SpF01_sRNA	
RNaseP_bact_b	SpF39_sRNA	
sau-50	SpF41_sRNA	
Spd-sr37		
SpR19_sRNA		
SSRC34		
tmRNA		
TPP		
tracrRNA		

Amongst the sRNA families overlapping with Core regions, FMN, L10_leader, Lacto-rpoB, PreQ1, Spd-sr37, tmRNA, and tracrRNA presented transcriptional signals, i.e., the probable occurrence of promoter and terminator signals detectable in at least one strain. RNA families RatA, rli28 and rli38 were found in Exclusive and Shared regions. Many of these sRNAs are known for their role in the regulation of metabolic processes, the biosynthesis of compounds or other key cellular processes (Additional file 5). Furthermore, RNA-OUT, SpF01_sRNA, SpF39_sRNA and SpF41_sRNA were found only in Exclusive regions, whereas sRNAs PyrR, SSRC38, TPP and yjdF were found in Shared regions.

**Fig. 2:**
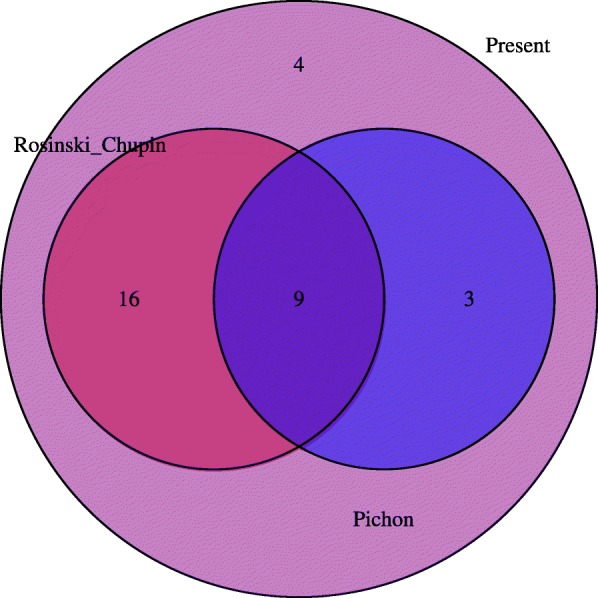
Number of ncRNAs detected in previous studies. Venn diagram shows the number of ncRNAs predicted that already have been detected on previous studies of [21, 22]

Noteworthy, while 28 Rfams reported by previous studies (from [21, 22]) were found in strain NEM316, the present analysis also led us to the identification of 4 new RNA families (SSRC38, rli38, sau-50 and SSRC34) in NEM316 Core region (Fig. 2**)**. An annotation file with new and previous information/data is provided as supplemental material (Additional file 4). Coverage plots based on mapped RNA-Seq data showed that families SSRC38, rli38 and SSRC34 seem to be transcribed (Additional file 8). Further studies are necessary to validate the functional role of these RNAs. The sau-50 family overlaps with a DNA binding protein and its activity was previously validated in another bacterial species [44], but unfortunately no additional information is available on its role and there is no noticeable difference in coverage by observing the transcriptome (Additional file 8).

**Fig. 3:**
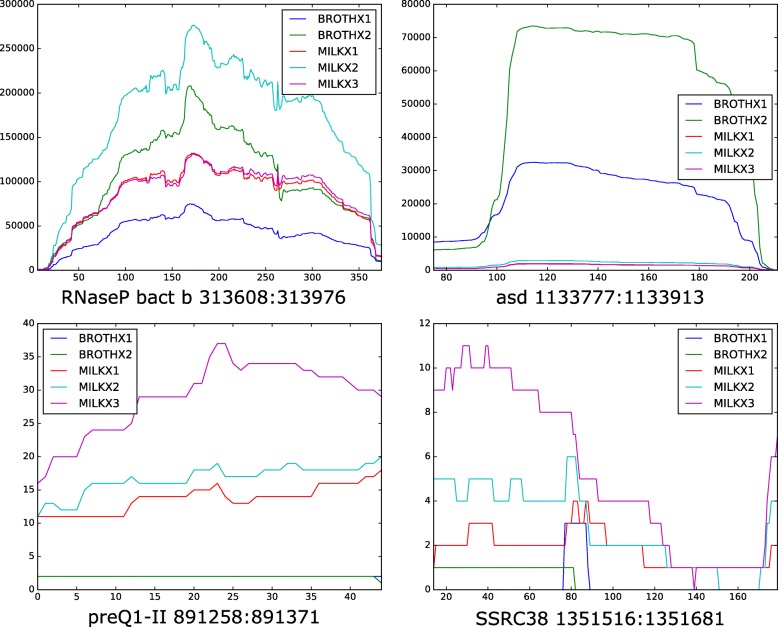
Coverage plots showing transcription activity for a sample of ncRNAs annotated. X Axis: annotated region; Y axis: raw coverage per base. RNaseP bact b is a ribozyme, *asd* is a intergenic non-coding RNA on NEM316, preQ1-II is a riboswitch and SSRC38 is a small RNA

Regarding the sRNA families found in Exclusive or Shared regions, many of them have not been characterized yet. Several of them were proposed as elements involved in the bacterial virulence (rli28, rli38 and SpF41), while others seemed to be related to mobile genetic elements (RatA and RNAout). The coverage plots based on mapped RNA-Seq data showed transcription activity for the remaining Rfams annotated (Fig. 3, coverage plots and read counts for all Rfams annotated are available on Additional file 9, additional coverage plots for a range of 800 and 1600 bp surrounding area of Rfam annotations is provided as Additional file 10). Although the number of raw reads (Y axis of Fig. 3 and Additional file 8) does not necessarily reflect the exact number of transcribed molecules, these plots provide supporting information on the transcription and potential role of the ncRNAs described in this study.

We last evaluated the clonal relationship of the strains included in this study by MLST analysis. This analysis showed large variability among isolates, which have little correlation with Rfams occurrence (Table 1).

## Discussion

The genomic regions found in GBS genomes are in accordance with the original description, which indicates that this genus has a Core genome and some regions without corresponding alignments in other strains [45, 46]. The observed overlap of several Shared and Exclusive regions with those probably acquired by horizontal gene transfer (HGT) (Fig. 1, tracks 4, 5 and 6) suggests that GBS genomes are continuously evolving, adapting and acquiring genetic information. Evidences of genome dynamics has been already reported in *S. agalactiae* by Brochet et al. [47].

RNA families annotation in the strain NEM316 showed that 88% were already reported by previous works ([21, 22]). The visualization of RNA-Seq data from Richards et al. [28] in the strain NEM316 also identified the transcriptional activity of the RNA families found in intergenic regions (e.g. Asd family in Fig. 3). However, our study resulted in the identification of 4 additional RNA families (SSRC38, rli38, sau-50 and SSRC34) (Fig. 2). Little is known in the function of these RNAs, notwithstanding transcription of SSRC38 and SSRC34 have been detected during exponential growth phase in *S. pyogenes* [48]. On the other hand, studies on rli38 function suggest that this RNA plays a key role in the pathogenesis of *Listeria monocytogenes* [49]. Last, although sau-50 have been reported as transcribed in other bacteria [44, 50] it still unclear the function of this RNA.

Although the annotated Rfams were not experimentally validated, transcriptomic data had already been used to successfully identify non coding RNAs (see [51, 52]). Furthermore, the annotation method utilized have already proved to be a powerful tool for large-scale analyses even in the presence of deletions, alterations in G + C content, and in distantly-related genomes [53]. Although not all annotated families show a significant coverage pattern, transcription is occurring in their loci. A possible explanation is that these RNAs are either co-transcribed or transcribed in different growth conditions. In either case they have the potential to act as regulatory elements [54]. As the annotation criteria for all strains were the same, the data obtained here represent a useful set of ncRNAs of *S. agalactiae*.

The comparative analysis in *S. agalactiae* genomes allowed us to detect 23 RNA families in Core regions and 17 (considering each Rfam database entry of group II introns) in the Shared and Exclusive regions (Table 2). However, the number of sRNA families was 25 in Core regions, while the sum of the Shared and Exclusive Rfams was greater than 28 for all cluster2 strains (Table 1) which give us a 25/28+ ratio of core/variable regions. This shows that HGT probably play a key role in the acquisition of ncRNAs as observed in *Salmonella* (see [54, 55]). Interestingly this was opposite to what was reported for *Escherichia coli* and *Shigella spp*. These bacteria have 60/23 ratio of core/variable regions [56]. It seems that Core and Shared regions in GBS genome evolved divergently for ncRNAs, where core regions are more conserved for the presence of these elements while shared regions provide the context for acquiring novel ncRNAs that have an adaptive benefit to the host.

We observed a strong correlation between the genome size and RNA families number, this direct relationship already has been observed in prokaryotes [53], and may explain the general variation of sRNAs when different strains of *S. agalactiae* are compared.

Cluster analysis determined three distinct clusters of *S. agalactiae*: cluster 2, fish and mammalian strains; cluster1, mammalian strains; and cluster0, fish strains (Table 1). The clusters resemble phylogenetic relationships revealed by single nucleotide polymorphism (SNP) analysis found in Rosinski-Chupin et al. [46] where CC7 is related to fish/human, CC19, CC23 and CC67 related to human/bovine and ST261, ST552 and ST260 to fish/frog. In addition the number of RNA families seems to be the factor that better separated the observed groups (Additional file 6). This finding provides evidence that there is a pattern concerning to strains identification based on Rfams number, therefore contributing to future research directions in the attempt to understand non-coding RNAs mechanisms and development of molecular markers.

All sRNAs with possible promoter and terminator transcriptional signals were located in the Core region overlapping the intergenic regions. Although the presence of these transcriptional signals increases the chance that Rfams are functional transcripts, they were not detected for some RNA families. This could be either due to the difficulty in finding such signals in intergenic regions, or because ncRNA promoter signals may be weak compared to the coding gene signals [57]. All signals found are tagged on annotation files provided as Additional file 4.

### RNA families in shared and exclusive regions

This in silico analysis led to the identification of two main ribozymes commonly found in bacteria, type B Ribonuclease P (RNase P) and group II (GII) introns. RNase P was found in all GBS genomes as expected since it is considered ubiquitous in bacteria. Its role is to catalyze the cleavage of the 5′-end of precursor transfer RNAs in order to generate the mature molecule [58, 59].

On the other hand, GII introns are ribozymes that catalyze their own splicing and in the presence of its cofactor became mobile by way of a retrotransposition mechanism [60]. All GII introns belonged to the subclass C and shared the same lineage with GBSi1 from *S. agalactiae* [61]. In their work the authors proposed that GBSi1 shares the same insertion site with the insertion sequence IS*1548* thus limiting its dissemination, and that this ribozyme may act as a marker for some clonal lineages of GBS. Our results are in line with the previous findings, since these elements were found in many but not all genomes (*n* = 10). However, we did not observe a correlation between the presence of this ribozyme and the clonal relationship (Additional file 5). Almost all GII introns had their cofactor, the intron encoded protein, which indicates that these ribozymes are active retroelements. Seven genomes (strains GD201008–001, HN016, YM001, GX064, GBS1-NY, GBS2-NM, GBS6) have a range from 9 to 19 non-redundant copies whereas the remaining 3 (strains 2603 V/R, NGBS572, GBS85147) only showed less than 3 copies. This indicates that when a genome contains the target site the ribozyme will invade it. Furthermore we observed that along GBS genomes there is a significant variation on ribozyme presence, amount of copies and target site availability. Taken together the data suggests that GII introns are key elements in the evolution and plasticity of GBS genomes.

Our results also showed that there is an association between the rli28 and RatA elements (Additional file 11). Genomes carrying both elements showed that there were 60 bp apart. It has been proposed that rli28 has a role in virulence [49], whereas RatA has been proposed as the antitoxin of TxpA toxin. Together RatA and TxpA act as type I toxin/antitoxin (TA) system [62]. Since this TA system has been described as located in phage-like elements, we evaluated whether this was also the case. Predictions with Phaster and Alienhunter confirmed that both rli28 and RatA are located in phages and therefore has the potentiality to be transferred to other bacterial species.

It is worth to mention that SSRC38 has been found in almost all genomes from clinical isolates, except for GBS85147 (Additional file 5). Although its function has not been yet described but its transcription reported in *S. pyogenes* [48], this sRNA seems to be an interesting candidate to be used as a molecular marker in the identification of new isolates or clones.

The presence of sRNAs in both Shared and Exclusive regions seems to be related to genomic areas with hotspots of recombination and insertion of mobile genetic elements. Frequently, phages can contribute to the creation of new interaction systems between hosts and pathogens [63] and regulatory RNAs can also be transferred between bacterial genomes [64]. Also, recombination between genomic sequences or sequences originating from phages may alter bacterial genomic structures so as to generate or eliminate RNA families [55].

### Families in Core regions

Almost all Rfams detected in Core regions were overlapping the intergenic regions. This predominance had previously been observed (see [65]). The only exception was sau-50 which is antisense in relation to a DNA-binding protein. It is worth to mention that sau-50 has been validated in *Staphylococcus aureus* [44] and also overlaps with a DNA binding protein.

RNA families related to essential activities and housekeeping functions were found in Core regions. Families like L10_leader control ribosomal protein transcription [66], FMN riboswitch is related to riboflavin production [67, 68], and tmRNAs release ribosomes caught in messenger RNAs without stop codons [69]. Clustered regularly interspaced short palindromic repeats (CRISPR) provide immunity against mobile genetic elements and had been analyzed in various strains of *S. agalactiae* (for details see [70]). The tracrRNA family, which is fundamental to the maturation of the CRISPR’s crRNAs [71] was found in Core regions.

Other RNA families found in the Core regions seems to be related with their upstream or downstream genes like 23S-Methyl, Lacto-rpoB and Spd-sr37 (as can be seen in [72–74] respectively).

Gene ontology (GO) terms associated to antibiotic resistance and cell membrane were observed in GBS host pathogen interactions [28]. sRNAs like Lacto-rpoB [73]) and Small Bacterial SRPs [75] also seems to be related to the previously cited GOs, thus these pieces of evidence suggest a putative relationship between sRNAs with GBS virulence, and thus can contribute with its pathogenicity.

## Conclusions

The original concept of pan-genome proposed by Tettelin et al. [45] for GBS was predicted on protein sequences. Since non-coding RNAs do not have a coding region they have been left aside.

*S. agalactiae* shows a compact core genome with few sRNAs at Core regions and various Rfams at its Exclusive and Shared regions. Thus, given the high recombination rate [76] and flexible gene pool of *S. agalactiae* [47], new non-coding RNAs are likely to continue to be discovered in modular genome of this species. Lastly, the existence of an open pan-genome for GBS [77] can be reaffirmed and could be expanded to encompass the full range of functional transcripts.

## Additional files

Additional file 1: Strain Information Table. (XLS 37 kb)

Additional file 2: Rfam entries only found in Lactobacillales order, used to annotation of ncRNAs with Infernal software. (TXT 1 kb)

Additional file 3: Curated ncRNA count. (XLSX 20 kb)

Additional file 4: GFF3 annotations for all classified Regions and ncRNAs. (ZIP 1638 kb)

Additional file 5: RNA families group, function and conservation information. (XLSX 20 kb)

Additional file 6: Plots of Features vs Cluster Assignments obtained on Weka by EM algorithm. (PDF 16 kb)

Additional file 7: Circos plots for all individual GBS genomes analyzed. All Additional file 4 information is plotted over genome extension. The tracks and the color code follow the same pattern as in Fig. 1. (ZIP 10957 kb)

Additional file 8: Coverage plots for surrounding region of new detected RNA families in strain NEM316. (PDF 1039 kb)

Additional file 9: Coverage plots and read counts for annotated ncRNAs regions of NEM316 strain. X axis on images are annotated region and Y axis are coverage per base. (ZIP 272 kb)

Additional file 10: Coverage plots for surrounding region of detected RNA families. (ZIP 2654 kb)

Additional file 11: rli and RatA RNA families counts and phage association information. (DOCX 59 kb)

## Abbreviations

CRISPR: Clustered regularly interspaced short palindromic repeats
crRNAs: CRISPR RNAs
dRNA-Seq: differential RNA-Seq data
EM: expectation-maximization
GBS: Group B Streptococcus
GII: group II introns
GO: Gene ontology
HGT: horizontal gene transfer
MLST: Multilocus sequence typing
ncRNA: non-coding RNA
Rfam: RNA Families
RNA: ribonucleic acid
RNase P: type B Ribonuclease P
RNA-Seq: RNA Sequencing
rRNA: ribossomic RNA
SRA: Sequence Read Archive
sRNA: Small RNA
TA: type I toxin/antitoxin system
tracrRNA: trans-activating crRNAs

## References

[1] Larsen JW, Sever JL (2008). Group B Streptococcus and pregnancy: a review. Am J Obstet Gynecol.

[2] Brochet M, Couvé E, Zouine M, Vallaeys T, Rusniok C, Lamy M-C, Buchrieser C, Trieu-Cuot P, Kunst F, Poyart C, Glaser P (2006). Genomic diversity and evolution within the species Streptococcus agalactiae. Microbes Infect.

[3] Salvador R, Eckehardt E, Julio M, De Freitas C (2005). Isolation and characterization of Streptococcus spp. group B in Nile tilapias (Oreochromis niloticus) reared in hapas nets and earth nurseries in the northern region of Parana state, Brazil. Ciência Rural.

[4] Mian GF, Godoy DT, Leal CA, GM YTYC, Figueiredo HC (2009). Aspects of the natural history and virulence of S. Agalactiae infection in Nile tilapia. Vet Microbiol.

[5] Li LP, Wang R, Liang WW, Huang T, Huang Y, Luo FG, Lei AY, Chen M, Gan X (2015). Development of live attenuated Streptococcus agalactiae vaccine for tilapia via continuous passage in vitro. Fish Shellfish Immunol.

[6] Yildirim A, Lämmler C, Weiß R (2002). Identification and characterization of Streptococcus agalactiae isolated from horses. Vet Microbiol.

[7] Elliott JA, Facklam RR, Richter CB (1990). Whole-cell protein patterns of nonhemolytic group B, type Ib, streptococci isolated from humans, mice, cattle, frogs, and fish. J Clin Microbiol.

[8] Di Palo B, Rippa V, Santi I, Brettoni C, Muzzi A, Metruccio MME, Grifantini R, Telford JL, Paccani SR, Soriani M (2013). Adaptive response of group B streptococcus to high glucose conditions: new insights on the CovRS regulation network. PLoS One.

[9] Mereghetti L, Sitkiewicz I, Green NM, Musser JM (2008). Remodeling of the Streptococcus agalactiae transcriptome in response to growth temperature. PLoS One.

[10] Mereghetti L, Sitkiewicz I, Green NM, Musser JM (2008). Extensive adaptive changes occur in the transcriptome of Streptococcus agalactiae (group B streptococcus) in response to incubation with human blood. PLoS One.

[11] Sitkiewicz I, Green NM, Guo N, Bongiovanni AM, Witkin SS, Musser JM (2009). Transcriptome adaptation of group B Streptococcus to growth in human amniotic fluid. PLoS One.

[12] Sorek R, Cossart P (2010). Prokaryotic transcriptomics: a new view on regulation, physiology and pathogenicity. Nat Rev Genet.

[13] Burge SW, Daub J, Eberhardt R, Tate J, Barquist L, Nawrocki EP, Eddy SR, Gardner PP, Bateman A (2013). Rfam 11.0: 10 years of RNA families. Nucleic Acids Res.

[14] Waters LS, Storz G (2009). Regulatory RNAs in bacteria. Cell.

[15] Toledo-Arana A, Repoila F, Cossart P (2007). Small noncoding RNAs controlling pathogenesis. Curr Opin Microbiol.

[16] Lenz DH, Mok KC, Lilley BN, Kulkarni RV, Wingreen NS, Bassler BL (2004). The small RNA chaperone Hfq and multiple small RNAs control quorum sensing in Vibrio harveyi and Vibrio cholerae. Cell.

[17] Sittka A, Lucchini S, Papenfort K, Sharma CM, Rolle K, Binnewies TT, Hinton JCD, Vogel J (2008). Deep sequencing analysis of small noncoding RNA and mRNA targets of the global post-transcriptional regulator, **Hfq**. PLoS Genet.

[18] Felden B, Vandenesch F, Bouloc P, Romby P (2011). The Staphylococcus aureus RNome and its commitment to virulence. PLoS Pathog.

[19] Patenge N, Pappesch R, Khani A, Kreikemeyer B (2015). Genome-wide analyses of small non-coding RNAs in streptococci. Front Genet.

[20] Le Rhun A, Beer YY, Reimegård J, Chylinski K, Charpentier E (2016). RNA sequencing uncovers antisense RNAs and novel small RNAs in Streptococcus pyogenes. RNA Biol.

[21] Pichon C, du Merle L, Caliot ME, Trieu-Cuot P, Le Bouguénec C (2012). An in silico model for identification of small RNAs in whole bacterial genomes: characterization of antisense RNAs in pathogenic Escherichia coli and Streptococcus agalactiae strains. Nucleic Acids Res.

[22] Rosinski-Chupin I, Sauvage E, Sismeiro O, Villain A, Da Cunha V, Caliot M-E, Dillies M-A, Trieu-Cuot P, Bouloc P, Lartigue M-F, Glaser P (2015). Single nucleotide resolution RNA-seq uncovers new regulatory mechanisms in the opportunistic pathogen Streptococcus agalactiae. BMC Genomics.

[23] Nawrocki EP, Burge SW, Bateman A, Daub J, Eberhardt RY, Eddy SR, Floden EW, Gardner PP, Jones TA, Tate J, Finn RD (2015). Rfam 12.0: updates to the RNA families database. Nucleic Acids Res.

[24] NCBI Resource coordinators (2013). Database resources of the National Center for biotechnology information. Nucleic Acids Res.

[25] Jolley KA, Maiden MC (2010). BIGSdb: scalable analysis of bacterial genome variation at the population level. BMC Bioinformatics.

[26] Darling AE, Mau B, Perna NT (2010). progressive mauve: multiple genome alignment with gene gain, loss and rearrangement. PLoS One.

[27] Glaser P, Rusniok C, Buchrieser C, Chevalier F, Frangeul L, Msadek T, Zouine M, Couvé E, Lalioui L, Poyart C, Trieu-Cuot P, Kunst F (2002). Genome sequence of Streptococcus agalactiae, a pathogen causing invasive neonatal disease. Mol Microbiol.

[28] Richards VP, Choi SC, Pavinski Bitar PD, Gurjar AA, Stanhope MJ (2013). Transcriptomic and genomic evidence for Streptococcus agalactiae adaptation to the bovine environment. BMC Genomics.

[29] Krzywinski M, Schein J, Birol I, Connors J, Gascoyne R, Horsman D, Jones SJ, Marra MA (2009). Circos: an information aesthetic for comparative genomics. Genome Res.

[30] Vernikos GS, Parkhill J (2006). Interpolated variable order motifs for identification of horizontally acquired DNA: revisiting the Salmonella pathogenicity islands. Bioinformatics.

[31] Dumas E, Christina Boritsch E, Vandenbogaert M, de la Vega RC R, Thiberge J-M, Caro V, Gaillard J-L, Heym B, Girard-Misguich F, Brosch R, Sapriel G (2016). Mycobacterial pan-genome analysis suggests important role of plasmids in the radiation of type VII secretion systems. Genome Biol Evol.

[32] Arndt D, Grant JR, Marcu A, Sajed T, Pon A, Liang Y, Wishart DS (2016). PHASTER: a better, faster version of the PHAST phage search tool. Nucleic Acids Res.

[33] Dhillon BK, Laird MR, Shay JA, Winsor GL, Lo R, Nizam F, Pereira SK, Waglechner N, McArthur AG, Langille MGI, Brinkman FSL (2015). IslandViewer 3: more flexible, interactive genomic island discovery, visualization and analysis. Nucleic Acids Res.

[34] Nawrocki EP, Eddy SR (2013). Infernal 1.1: 100-fold faster RNA homology searches. Bioinformatics.

[35] Nawrocki EP (2014). RNA sequence, structure, and function: computational and Bioinformatic methods. Volume 1097.

[36] Herbig A, Nieselt K (2011). nocoRNAc: characterization of non-coding RNAs in prokaryotes. BMC Bioinformatics.

[37] Rutherford K, Parkhill J, Crook J, Horsnell T, Rice P, Rajandream MA, Barrell B (2000). Artemis: sequence visualization and annotation. Bioinformatics.

[38] R Core Team (2015). R: A Language and Environment for Statistical Computing.

[39] Bolger AM, Lohse M, Usadel B (2014). Trimmomatic: a flexible trimmer for Illumina sequence data. Bioinformatics.

[40] Langmead B, Salzberg SL (2012). Fast gapped-read alignment with bowtie 2. Nat Methods.

[41] Li H, Handsaker B, Wysoker A, Fennell T, Ruan J, Homer N, Marth G, Abecasis G, Durbin R, 1000 genome project data processing subgroup (2009). The sequence alignment/map format and SAMtools. Bioinformatics.

[42] Yin T, Cook D, Lawrence M (2012). ggbio: an R package for extending the grammar of graphics for genomic data. Genome Biol.

[43] Hall M, National H, Frank E, Holmes G, Pfahringer B, Reutemann P, Witten IH (2009). The WEKA data mining Software : an update. SIGKDD Explor.

[44] Abu-Qatouseh LF, Chinni SV, Seggewiß J, Proctor RA, Brosius J, Rozhdestvensky TS, Peters G, Eiff C, Von BK (2010). Identification of differentially expressed small non-protein-coding RNAs in Staphylococcus aureus displaying both the normal and the small-colony variant phenotype. J Mol Med.

[45] Tettelin H, Masignani V, Cieslewicz MJ, Donati C, Medini D, Ward NL, Angiuoli SV, Crabtree J, Jones AL, Durkin AS, Deboy RT, Davidsen TM, Mora M, Scarselli M, Margarit y Ros I, Peterson JD, Hauser CR, Sundaram JP, Nelson WC, Madupu R, Brinkac LM, Dodson RJ, Rosovitz MJ, Sullivan SA, Daugherty SC, Haft DH, Selengut J, Gwinn ML, Zhou L, Zafar N (2005). Genome analysis of multiple pathogenic isolates of Streptococcus agalactiae: implications for the microbial “pan-genome”. Proc Natl Acad Sci U S A.

[46] Rosinski-Chupin I, Sauvage E, Mairey B, Mangenot S, Ma L, Da Cunha V, Rusniok C, Bouchier C, Barbe V, Glaser P (2013). Reductive evolution in Streptococcus agalactiae and the emergence of a host adapted lineage. BMC Genomics.

[47] Brochet M, Rusniok C, Couve E, Dramsi S, Poyart C, Trieu-Cuot P, Kunst F, Glaser P (2008). Shaping a bacterial genome by large chromosomal replacements, the evolutionary history of Streptococcus agalactiae. Proc Natl Acad Sci.

[48] Tesorero RA, Yu N, Wright JO, Svencionis JP, Cheng Q, Kim J-H, Cho KH (2013). Novel regulatory small RNAs in Streptococcus pyogenes. PLoS One.

[49] Toledo-Arana A, Dussurget O, Nikitas G, Sesto N, Guet-Revillet H, Balestrino D, Loh E, Gripenland J, Tiensuu T, Vaitkevicius K, Barthelemy M, Vergassola M, Nahori M-A, Soubigou G, Régnault B, Coppée J-Y, Lecuit M, Johansson J, Cossart P (2009). The Listeria transcriptional landscape from saprophytism to virulence. Nature.

[50] Boutard M, Ettwiller L, Cerisy T, Alberti A, Labadie K, Salanoubat M, Schildkraut I, Tolonen AC (2016). Global repositioning of transcription start sites in a plant-fermenting bacterium. Nat Commun.

[51] Beaume M, Hernandez D, Farinelli L, Deluen C, Linder P, Gaspin C, Romby P, Schrenzel J, Francois P (2010). Cartography of methicillin-resistant S. aureus transcripts: detection, orientation and temporal expression during growth phase and stress conditions. PLoS One.

[52] Irnov I, Sharma CM, Vogel J, Winkler WC (2010). Identification of regulatory RNAs in Bacillus subtilis. Nucleic Acids Res.

[53] Matelska D, Kurkowska M, Purta E, Bujnicki JM, Dunin-Horkawicz S (2016). Loss of conserved noncoding RNAs in genomes of bacterial endosymbionts. Genome Biol Evol.

[54] Papenfort K, Vogel J (2010). Regulatory RNA in bacterial pathogens. Cell Host Microbe.

[55] Raghavan R, Kacharia FR, Millar JA, Sislak CD, Ochman H (2015). Genome rearrangements can make and break small RNA genes. Genome Biol Evol.

[56] Skippington E, Ragan MA (2012). Evolutionary dynamics of small RNAs in 27 escherichia coli and shigella genomes. Genome Biol Evol.

[57] Sridhar J, Gunasekaran P (2013). Computational small RNA prediction in bacteria. Bioinform Biol Insights.

[58] Guerrier-Takada C, Gardiner K, Marsh T, Pace N, Altman S (1983). The RNA moiety of ribonuclease P is the catalytic subunit of the enzyme. Cell.

[59] Ellis JC, Brown JW (2009). The RNase P family. RNA Biol.

[60] Lambowitz AM, Zimmerly S (2011). Group II introns: mobile ribozymes that invade DNA. Cold Spring Harb Perspect Biol.

[61] Granlund M, Michel F, Norgren M (2001). Mutually exclusive distribution of IS1548 and GBSi1, an active group II intron identified in human isolates of group B streptococci. J Bacteriol.

[62] Silvaggi JM, Perkins JB, Losick R (2005). Small untranslated RNA antitoxin in Bacillus subtilis. J Bacteriol.

[63] Toft C, Andersson SGE (2010). Evolutionary microbial genomics: insights into bacterial host adaptation. Nat Rev Genet.

[64] Gottesman S, Storz G. Bacterial small RNA regulators: versatile roles and rapidly evolving variations. Cold Spring Harb Perspect Biol. 2011;3. 10.1101/cshperspect.a003798. 10.1101/cshperspect.a003798 PMC3225950 20980440

[65] Tsai C-H, Liao R, Chou B, Palumbo M, Contreras LM (2015). Genome-wide analyses in Bacteria show small-RNA enrichment for long and conserved intergenic regions. J Bacteriol.

[66] Zengel JM, Lindahl L (1994). Diverse mechanisms for regulating ribosomal protein synthesis in Escherichia coli. Prog Nucleic Acid Res Mol Biol.

[67] Vitreschak AG, Rodionov DA, Mironov AA, Gelfand MS (2002). Regulation of riboflavin biosynthesis and transport genes in bacteria by transcriptional and translational attenuation. Nucleic Acids Res.

[68] Serganov A, Huang L, Patel DJ (2009). Coenzyme recognition and gene regulation by a flavin mononucleotide riboswitch. Nature.

[69] Wower IK, Zwieb C, Wower J (2005). Transfer-messenger RNA unfolds as it transits the ribosome. RNA.

[70] Lopez-Sanchez M-J, Sauvage E, Da Cunha V, Clermont D, Ratsima Hariniaina E, Gonzalez-Zorn B, Poyart C, Rosinski-Chupin I, Glaser P (2012). The highly dynamic CRISPR1 system of Streptococcus agalactiae controls the diversity of its mobilome. Mol Microbiol.

[71] Deltcheva E, Chylinski K, Sharma CM, Gonzales K, Chao Y, Pirzada ZA, Eckert MR, Vogel J, Charpentier E (2011). CRISPR RNA maturation by trans-encoded small RNA and host factor RNase III. Nature.

[72] Weinberg Z, Barrick JE, Yao Z, Roth A, Kim JN, Gore J, Wang JX, Lee ER, Block KF, Sudarsan N, Neph S, Tompa M, Ruzzo WL, Breaker RR (2007). Identification of 22 candidate structured RNAs in bacteria using the CMfinder comparative genomics pipeline. Nucleic Acids Res.

[73] Goldstein BP (2014). Resistance to rifampicin: a review. J Antibiot (Tokyo).

[74] Tsui H-CT, Mukherjee D, Ray VA, Sham L-T, Feig AL, Winkler ME (2010). Identification and characterization of noncoding small RNAs in Streptococcus pneumoniae serotype 2 strain D39. J Bacteriol.

[75] Ulbrandt ND, Newitt JA, Bernstein HD (1997). The E-coli signal recognition particle is required for the insertion of a subset of inner membrane proteins. Cell.

[76] Da Cunha V, Davies MR, Douarre P-E, Rosinski-Chupin I, Margarit I, Spinali S, Perkins T, Lechat P, Dmytruk N, Sauvage E, Ma L, Romi B, Tichit M, Lopez-Sanchez M-J, Descorps-Declere S, Souche E, Buchrieser C, Trieu-Cuot P, Moszer I, Clermont D, Maione D, Bouchier C, DJ MM, Parkhill J, Telford JL, Dougan G, Walker MJ, Melin P, Decheva A, Petrunov B (2014). *Streptococcus agalactiae* clones infecting humans were selected and fixed through the extensive use of tetracycline. Nat Commun.

[77] Medini D, Donati C, Tettelin H, Masignani V, Rappuoli R (2005). The microbial pan-genome. Curr Opin Genet Dev.

