# Supplementary figures and images for: Functional annotation and distribution overview of RNA families in 27 Streptococcus agalactiae genomes
Source: BMC Genomics. 2018 Jul 28;19:556. doi: 10.1186/s12864-018-4951-z (PMC6064168; doi:10.1186/s12864-018-4951-z)

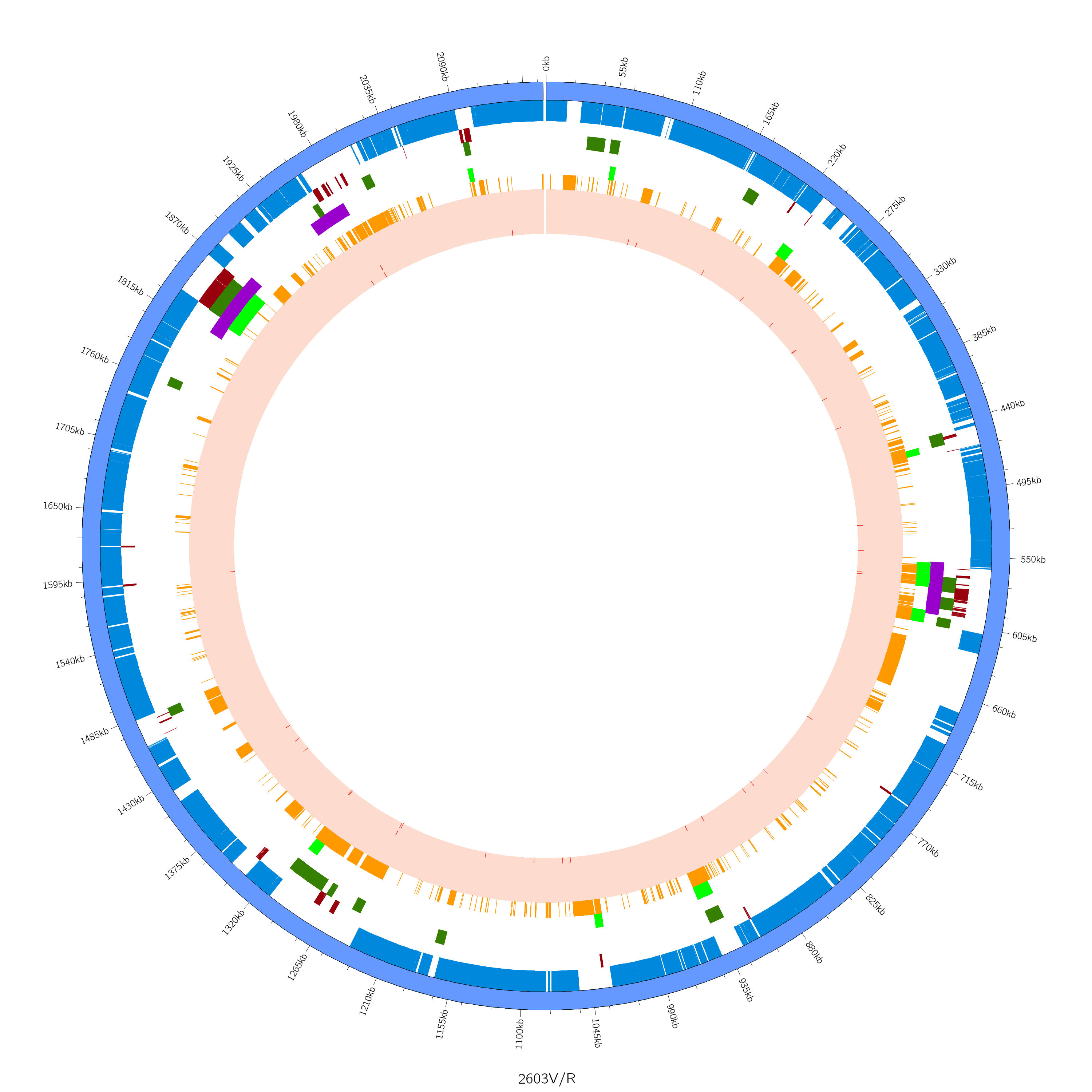

Supplement: Supplementary file 7 — Circos plots for all individual GBS genomes analyzed. All Additional file 4 information is plotted over genome extension. The tracks and the color code follow the same pattern as in Fig. 1. (ZIP 10957 kb) [file 12864_2018_4951_MOESM7_ESM.zip › Additional file 6/AE009948.png]

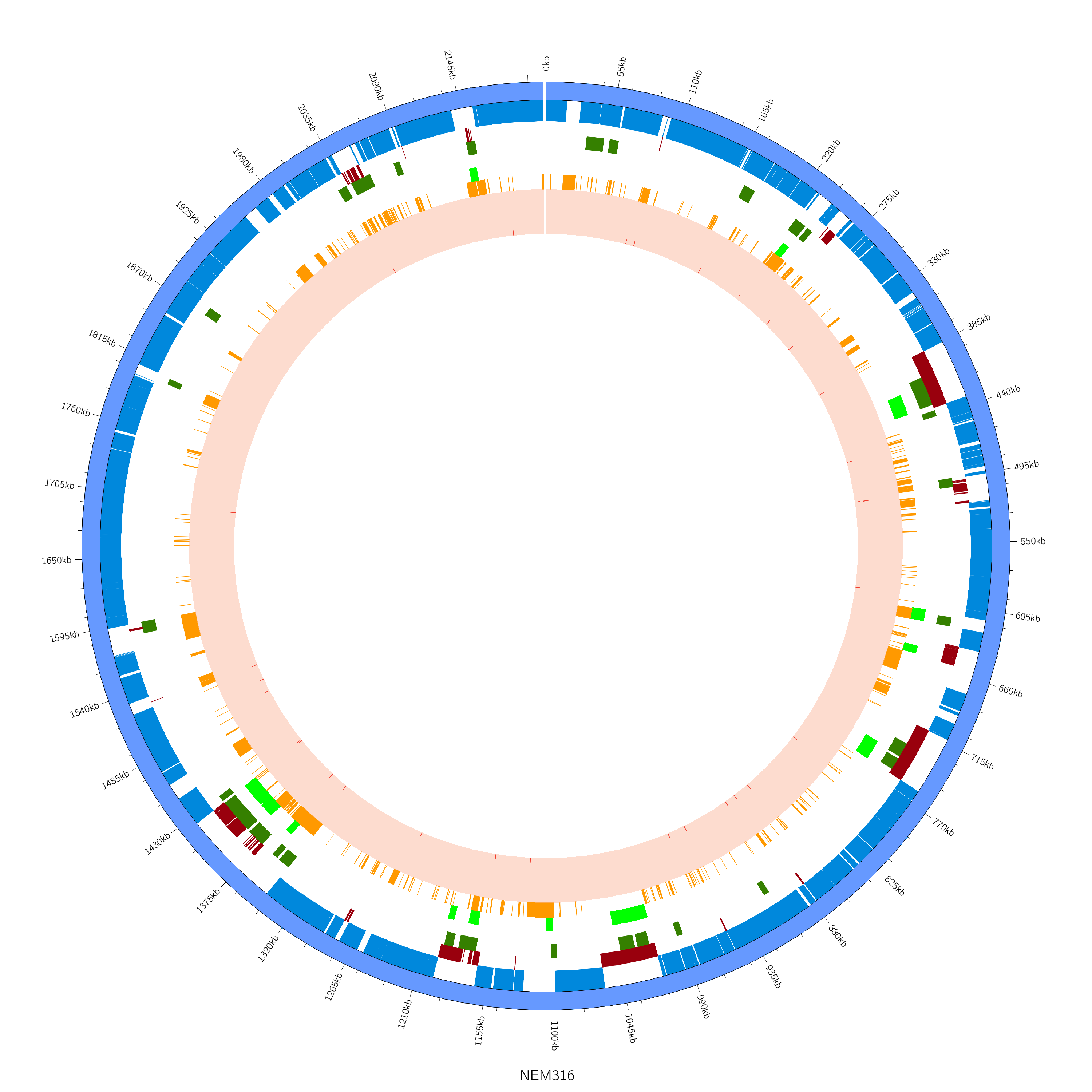

Supplement: Supplementary file 7 — Circos plots for all individual GBS genomes analyzed. All Additional file 4 information is plotted over genome extension. The tracks and the color code follow the same pattern as in Fig. 1. (ZIP 10957 kb) [file 12864_2018_4951_MOESM7_ESM.zip › Additional file 6/AL732656.png]

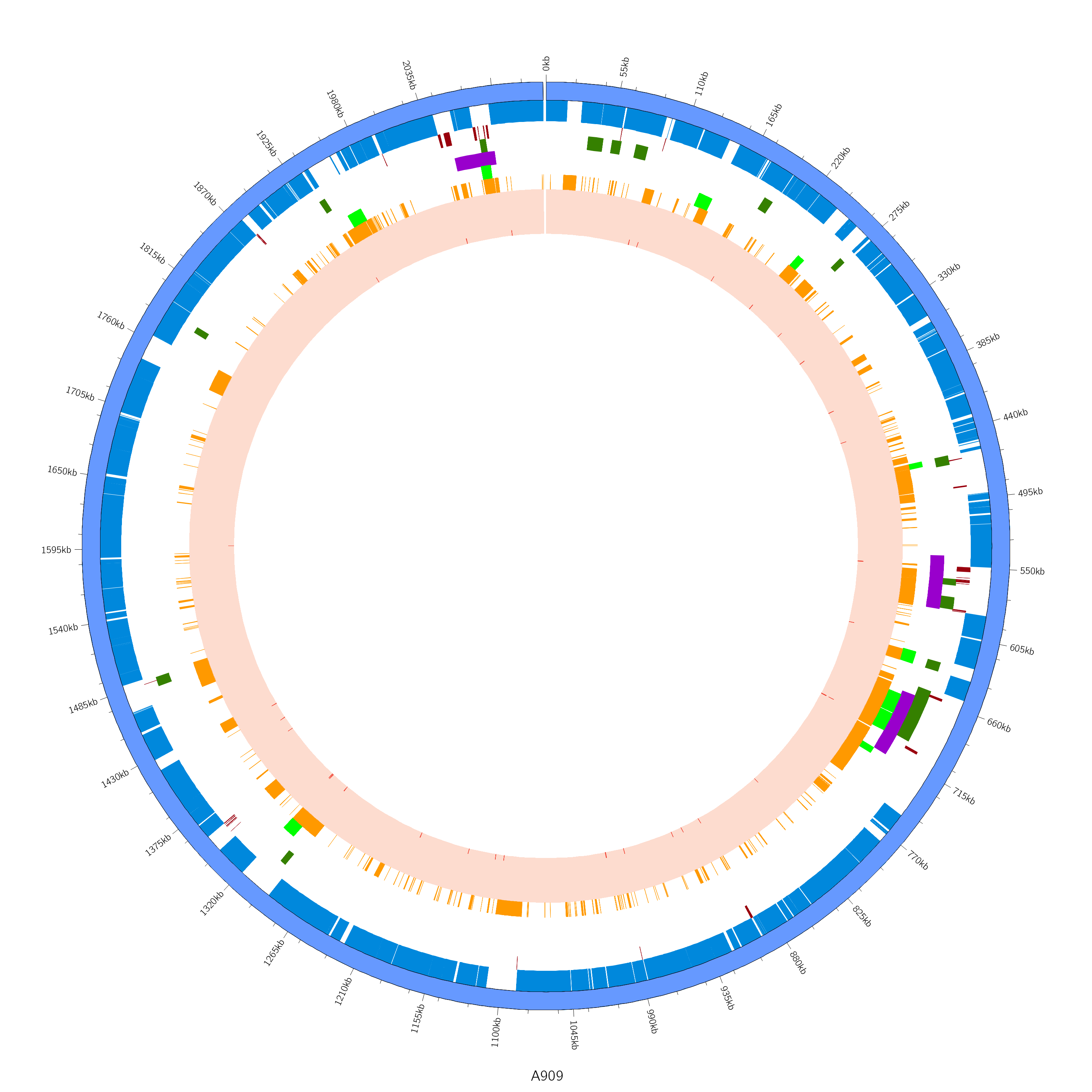

Supplement: Supplementary file 7 — Circos plots for all individual GBS genomes analyzed. All Additional file 4 information is plotted over genome extension. The tracks and the color code follow the same pattern as in Fig. 1. (ZIP 10957 kb) [file 12864_2018_4951_MOESM7_ESM.zip › Additional file 6/CP000114.png]

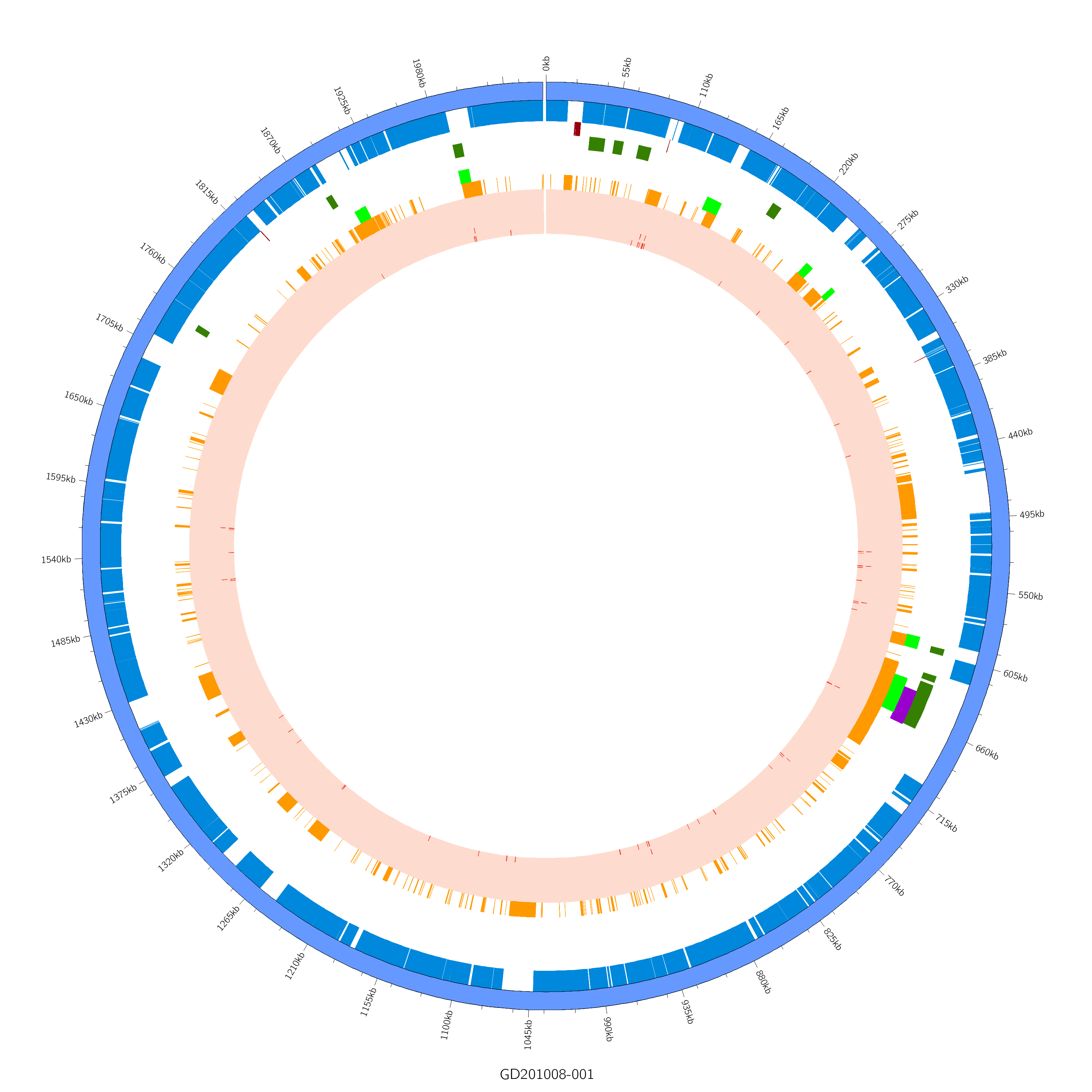

Supplement: Supplementary file 7 — Circos plots for all individual GBS genomes analyzed. All Additional file 4 information is plotted over genome extension. The tracks and the color code follow the same pattern as in Fig. 1. (ZIP 10957 kb) [file 12864_2018_4951_MOESM7_ESM.zip › Additional file 6/CP003810.png]

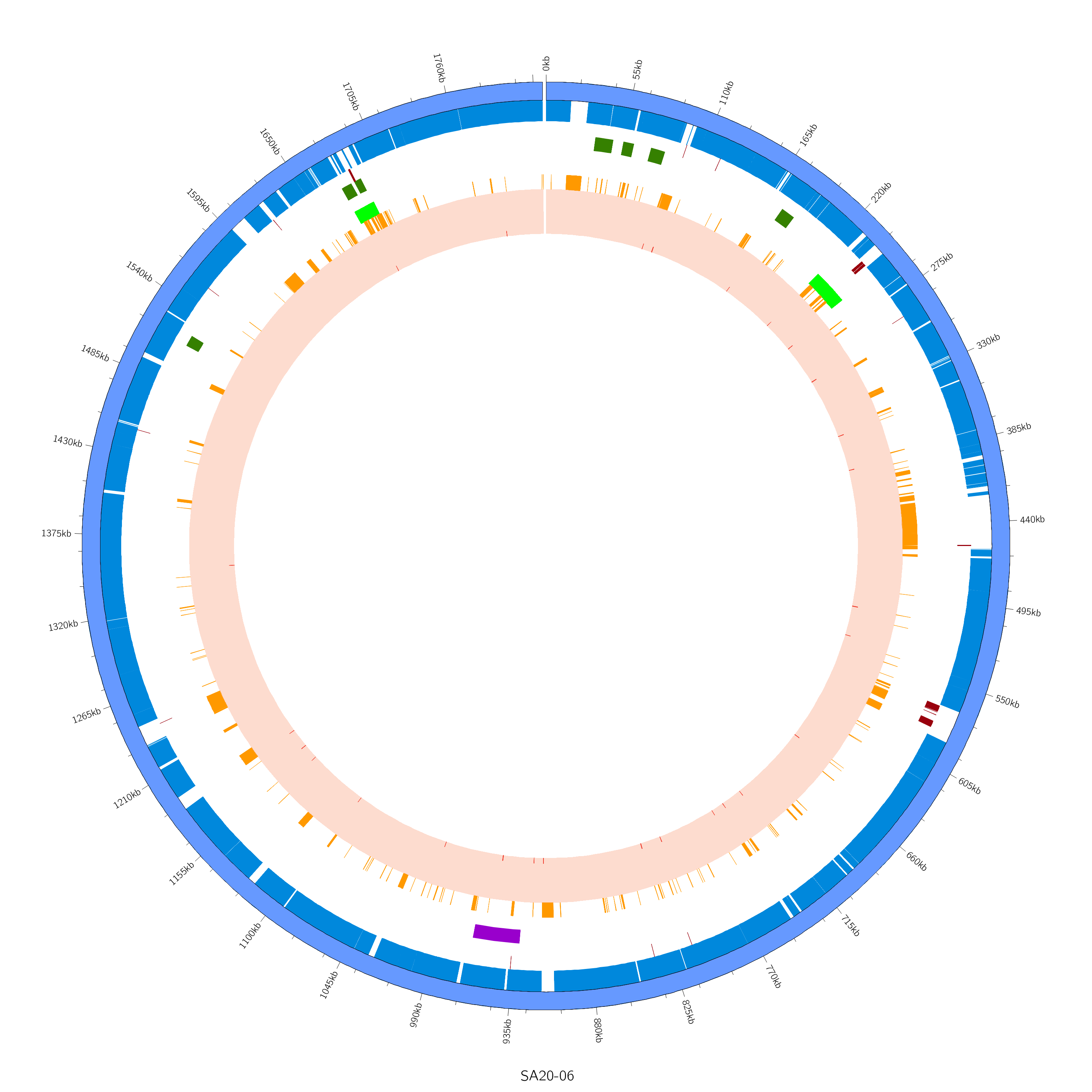

Supplement: Supplementary file 7 — Circos plots for all individual GBS genomes analyzed. All Additional file 4 information is plotted over genome extension. The tracks and the color code follow the same pattern as in Fig. 1. (ZIP 10957 kb) [file 12864_2018_4951_MOESM7_ESM.zip › Additional file 6/CP003919.png]

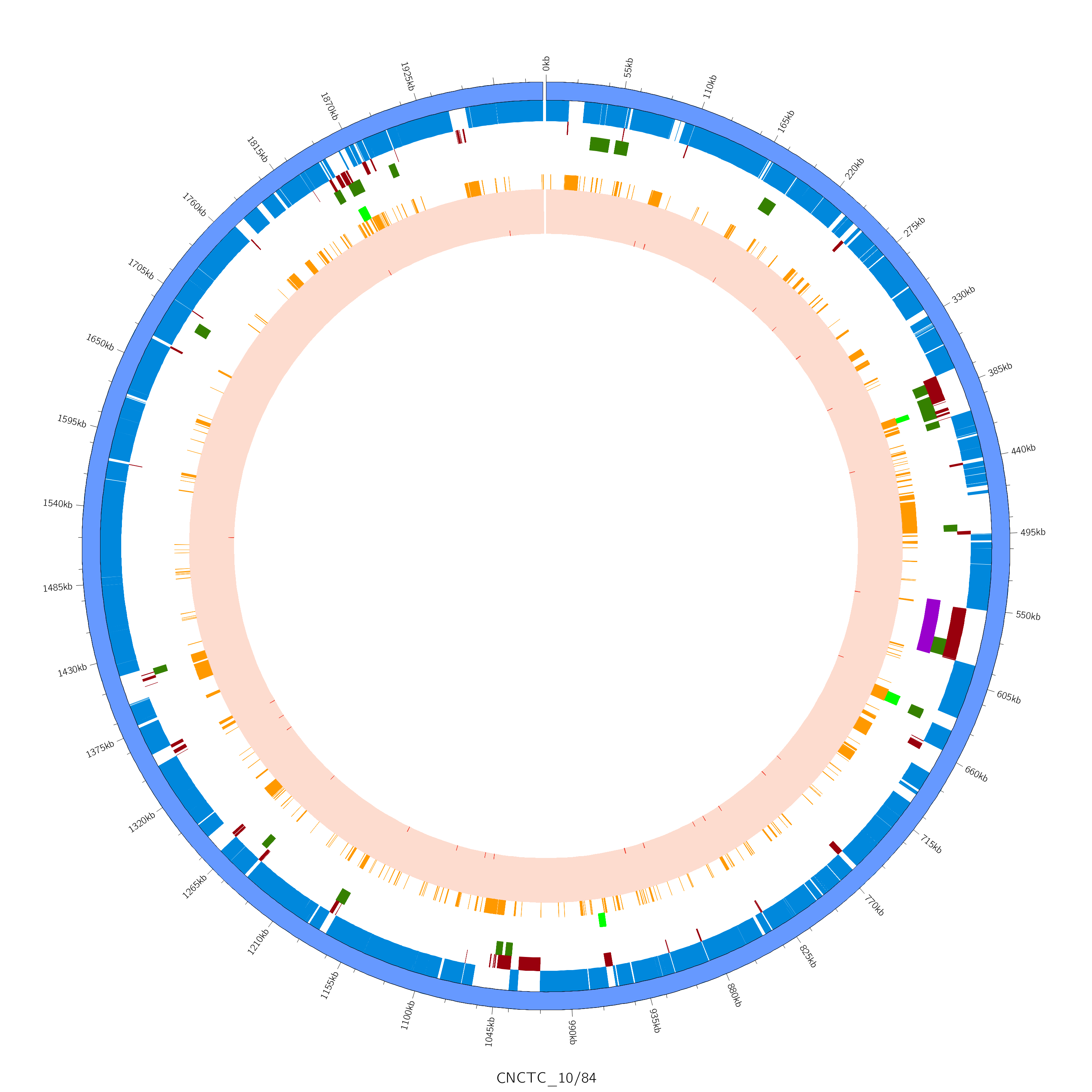

Supplement: Supplementary file 7 — Circos plots for all individual GBS genomes analyzed. All Additional file 4 information is plotted over genome extension. The tracks and the color code follow the same pattern as in Fig. 1. (ZIP 10957 kb) [file 12864_2018_4951_MOESM7_ESM.zip › Additional file 6/CP006910.png]

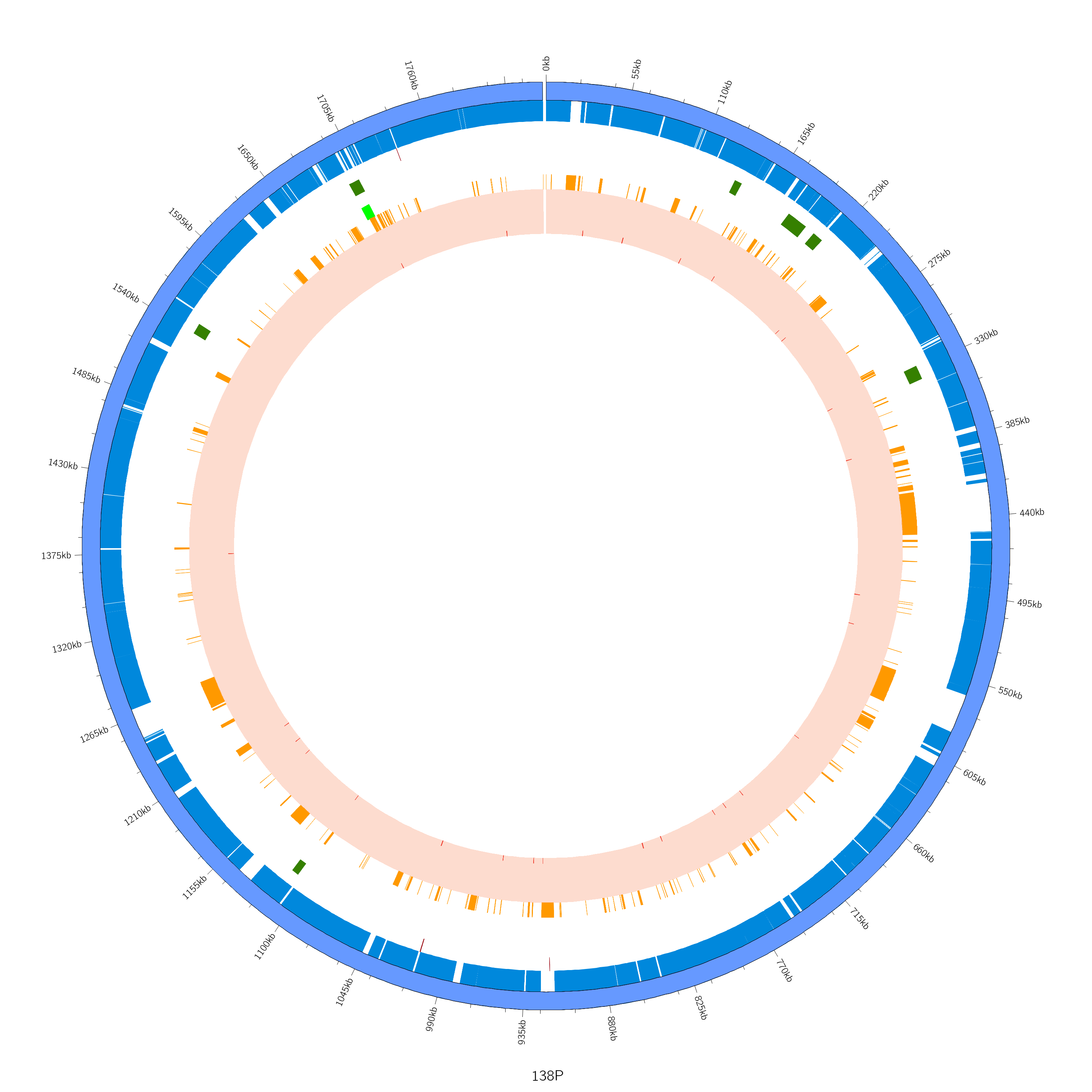

Supplement: Supplementary file 7 — Circos plots for all individual GBS genomes analyzed. All Additional file 4 information is plotted over genome extension. The tracks and the color code follow the same pattern as in Fig. 1. (ZIP 10957 kb) [file 12864_2018_4951_MOESM7_ESM.zip › Additional file 6/CP007482.png]

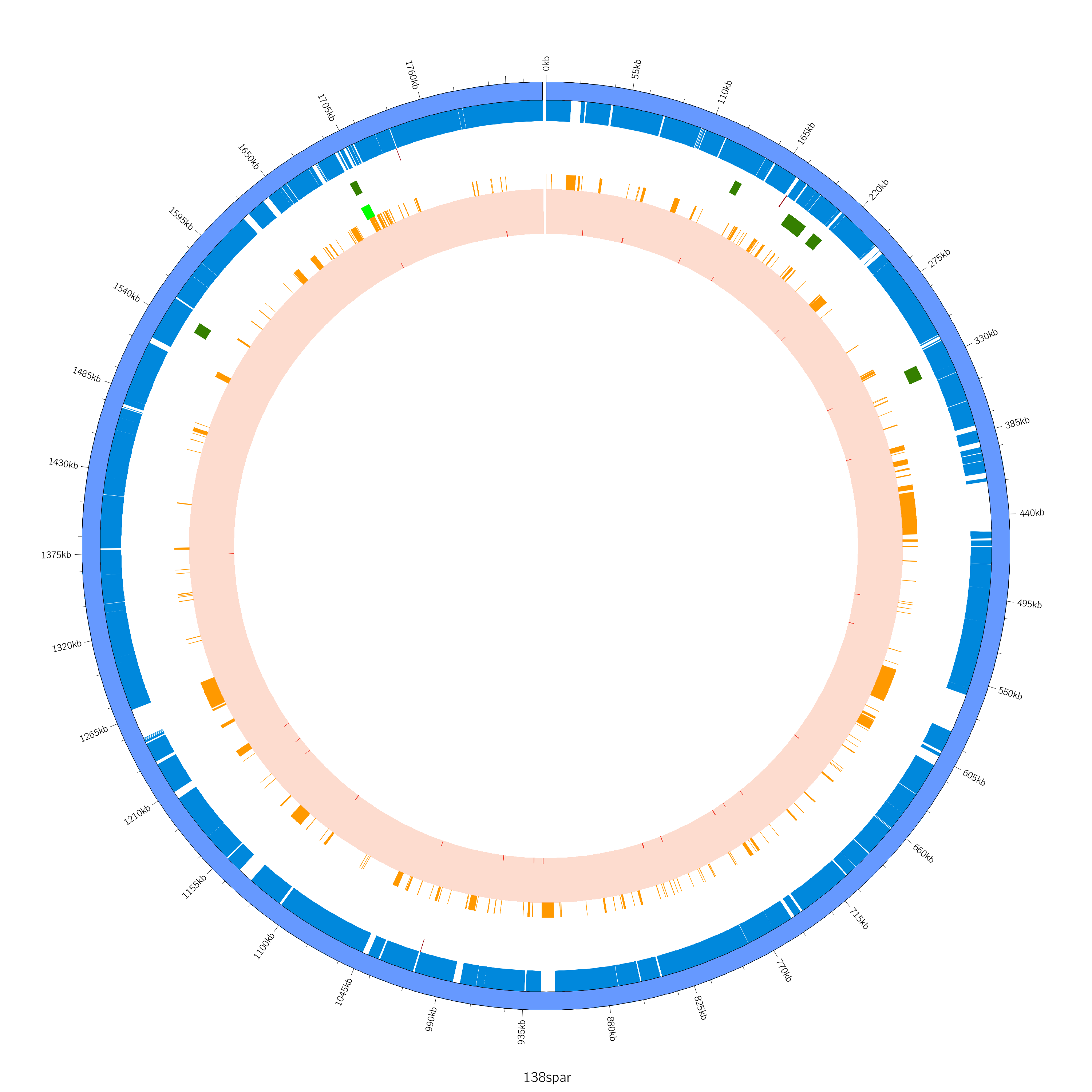

Supplement: Supplementary file 7 — Circos plots for all individual GBS genomes analyzed. All Additional file 4 information is plotted over genome extension. The tracks and the color code follow the same pattern as in Fig. 1. (ZIP 10957 kb) [file 12864_2018_4951_MOESM7_ESM.zip › Additional file 6/CP007565.png]

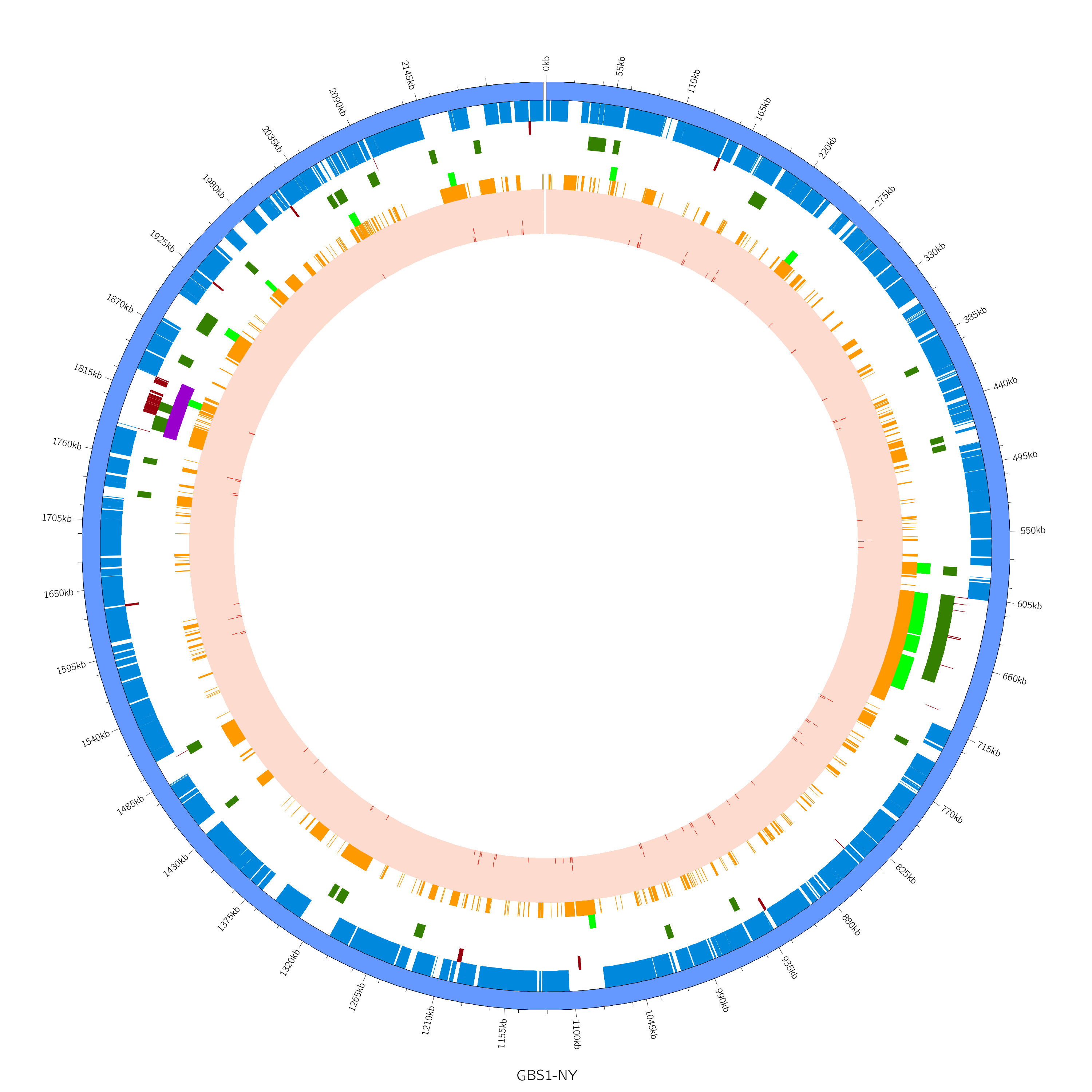

Supplement: Supplementary file 7 — Circos plots for all individual GBS genomes analyzed. All Additional file 4 information is plotted over genome extension. The tracks and the color code follow the same pattern as in Fig. 1. (ZIP 10957 kb) [file 12864_2018_4951_MOESM7_ESM.zip › Additional file 6/CP007570.png]

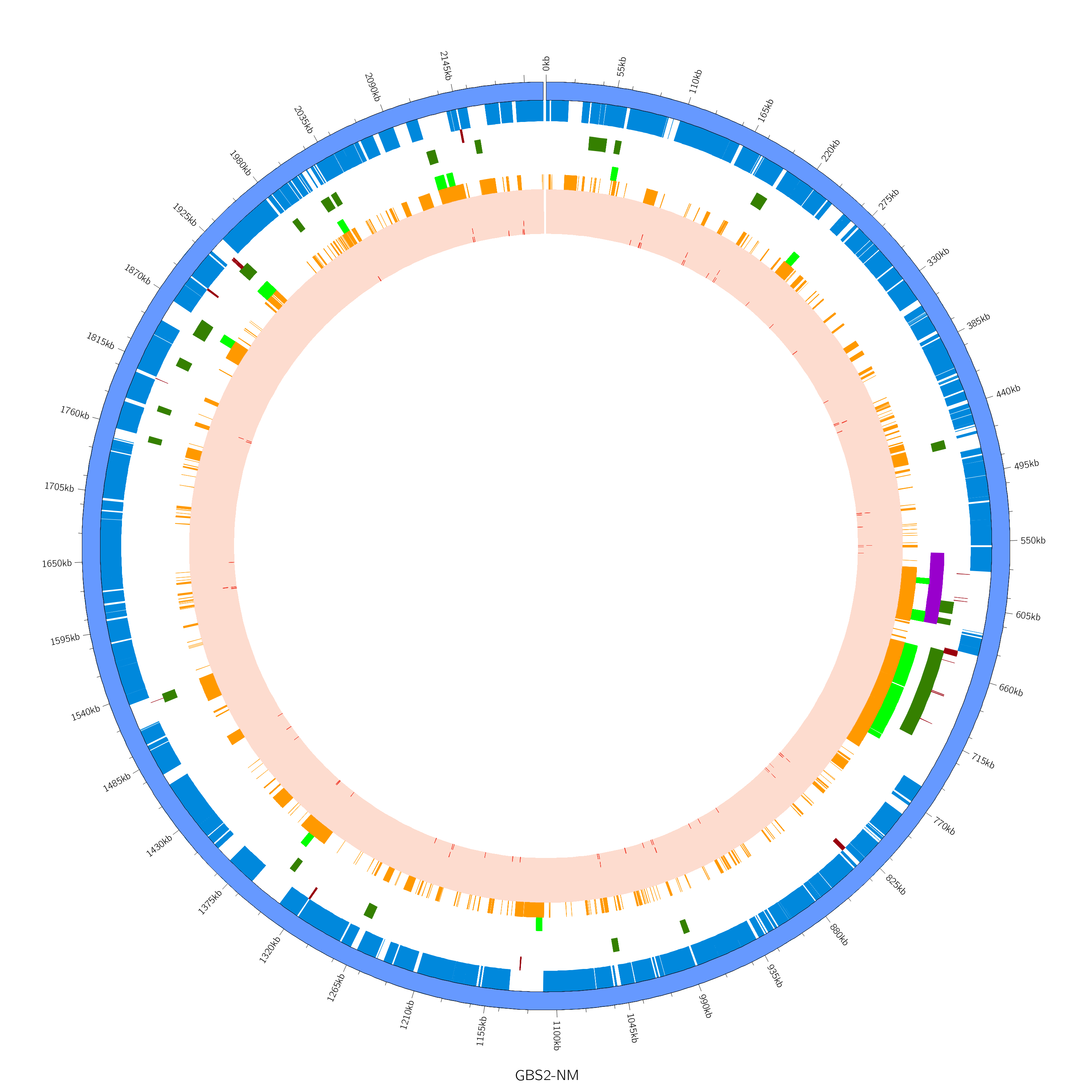

Supplement: Supplementary file 7 — Circos plots for all individual GBS genomes analyzed. All Additional file 4 information is plotted over genome extension. The tracks and the color code follow the same pattern as in Fig. 1. (ZIP 10957 kb) [file 12864_2018_4951_MOESM7_ESM.zip › Additional file 6/CP007571.png]

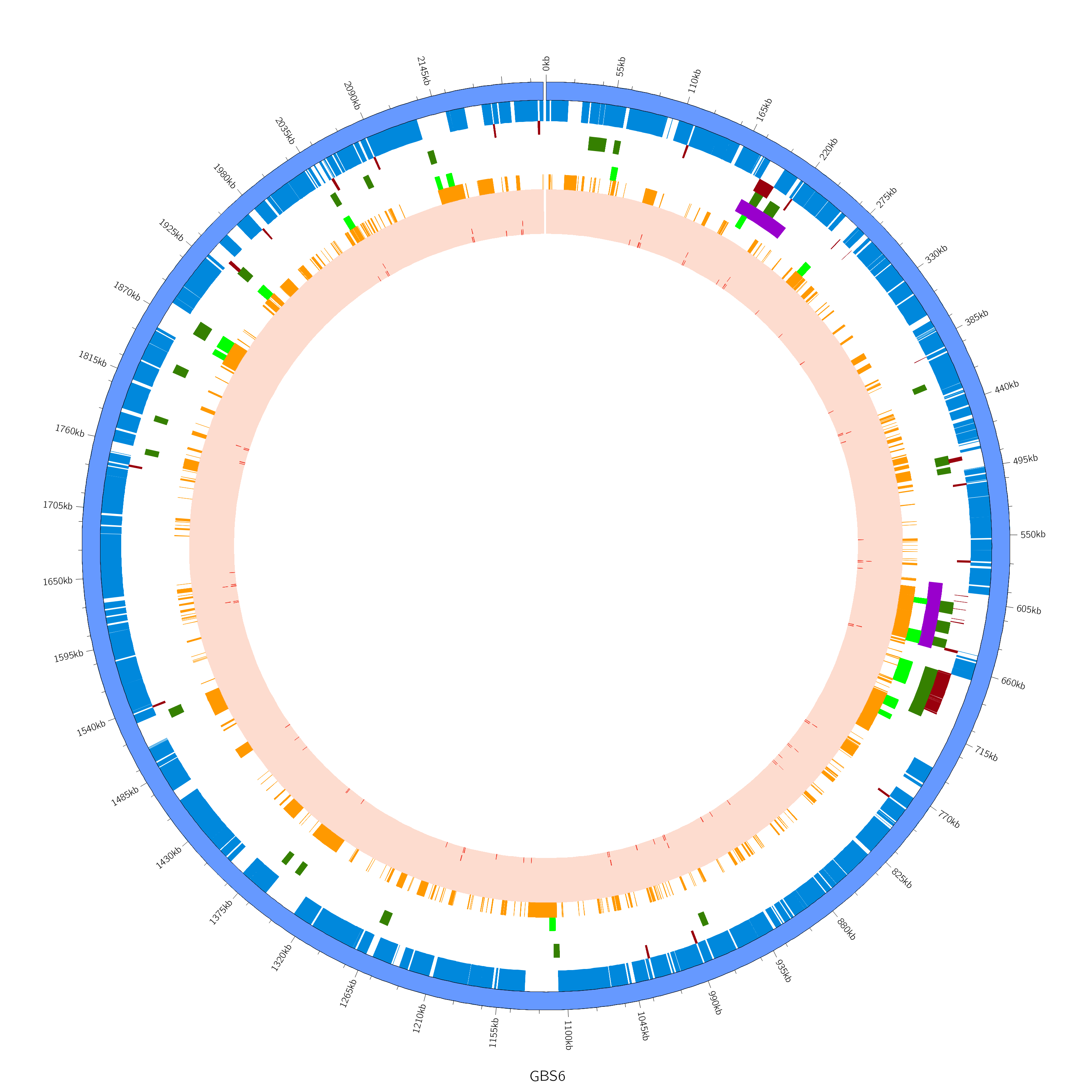

Supplement: Supplementary file 7 — Circos plots for all individual GBS genomes analyzed. All Additional file 4 information is plotted over genome extension. The tracks and the color code follow the same pattern as in Fig. 1. (ZIP 10957 kb) [file 12864_2018_4951_MOESM7_ESM.zip › Additional file 6/CP007572.png]

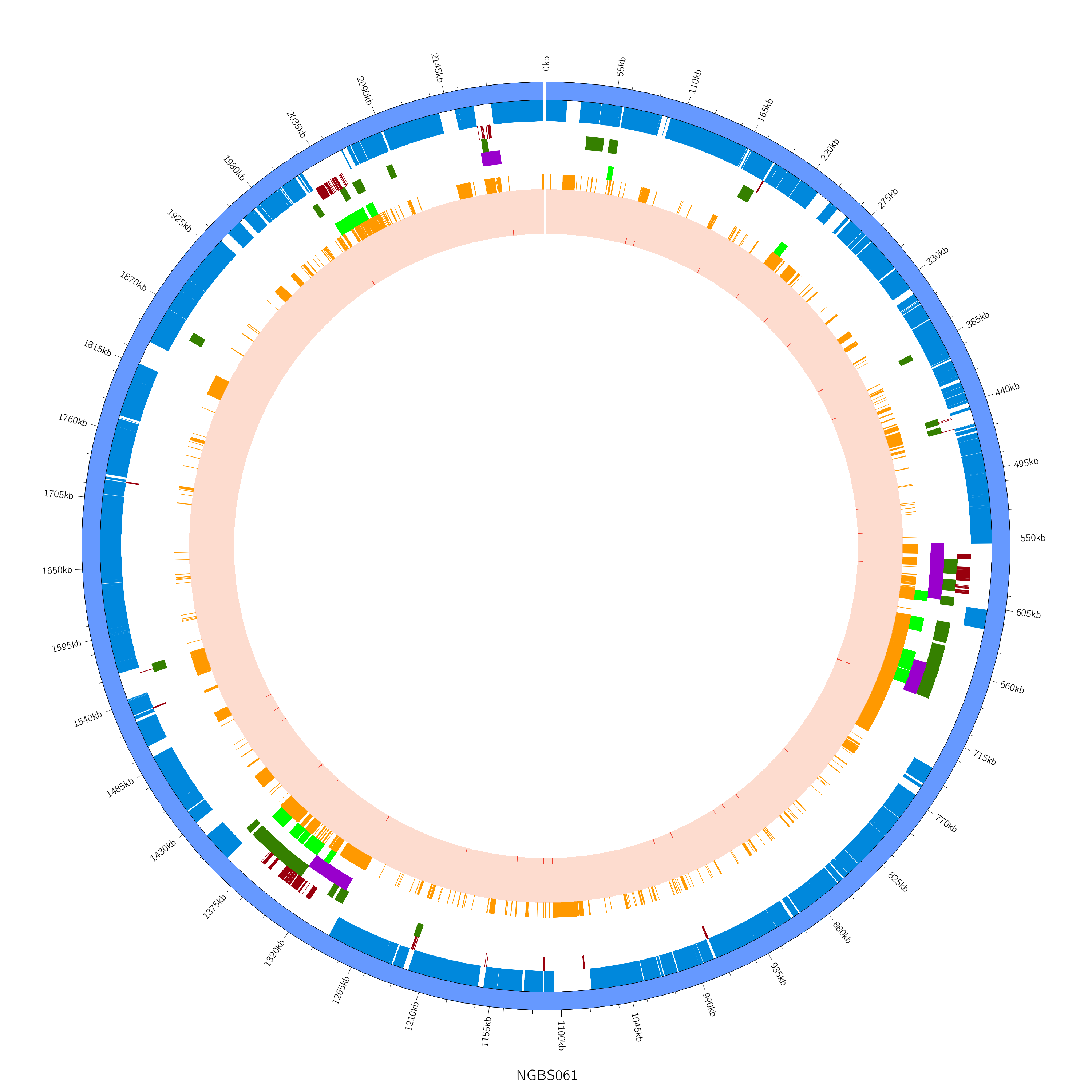

Supplement: Supplementary file 7 — Circos plots for all individual GBS genomes analyzed. All Additional file 4 information is plotted over genome extension. The tracks and the color code follow the same pattern as in Fig. 1. (ZIP 10957 kb) [file 12864_2018_4951_MOESM7_ESM.zip › Additional file 6/CP007631.png]

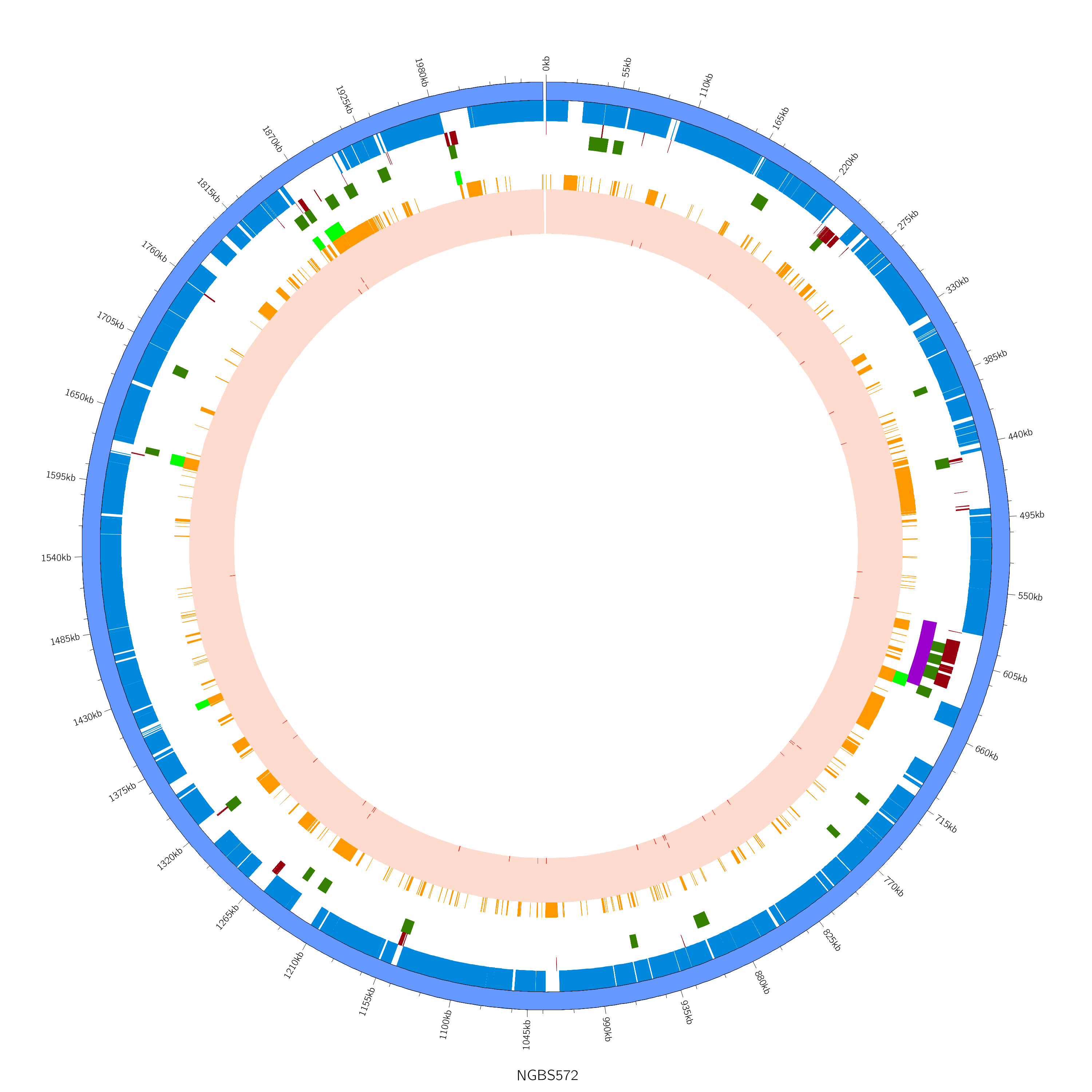

Supplement: Supplementary file 7 — Circos plots for all individual GBS genomes analyzed. All Additional file 4 information is plotted over genome extension. The tracks and the color code follow the same pattern as in Fig. 1. (ZIP 10957 kb) [file 12864_2018_4951_MOESM7_ESM.zip › Additional file 6/CP007632.png]

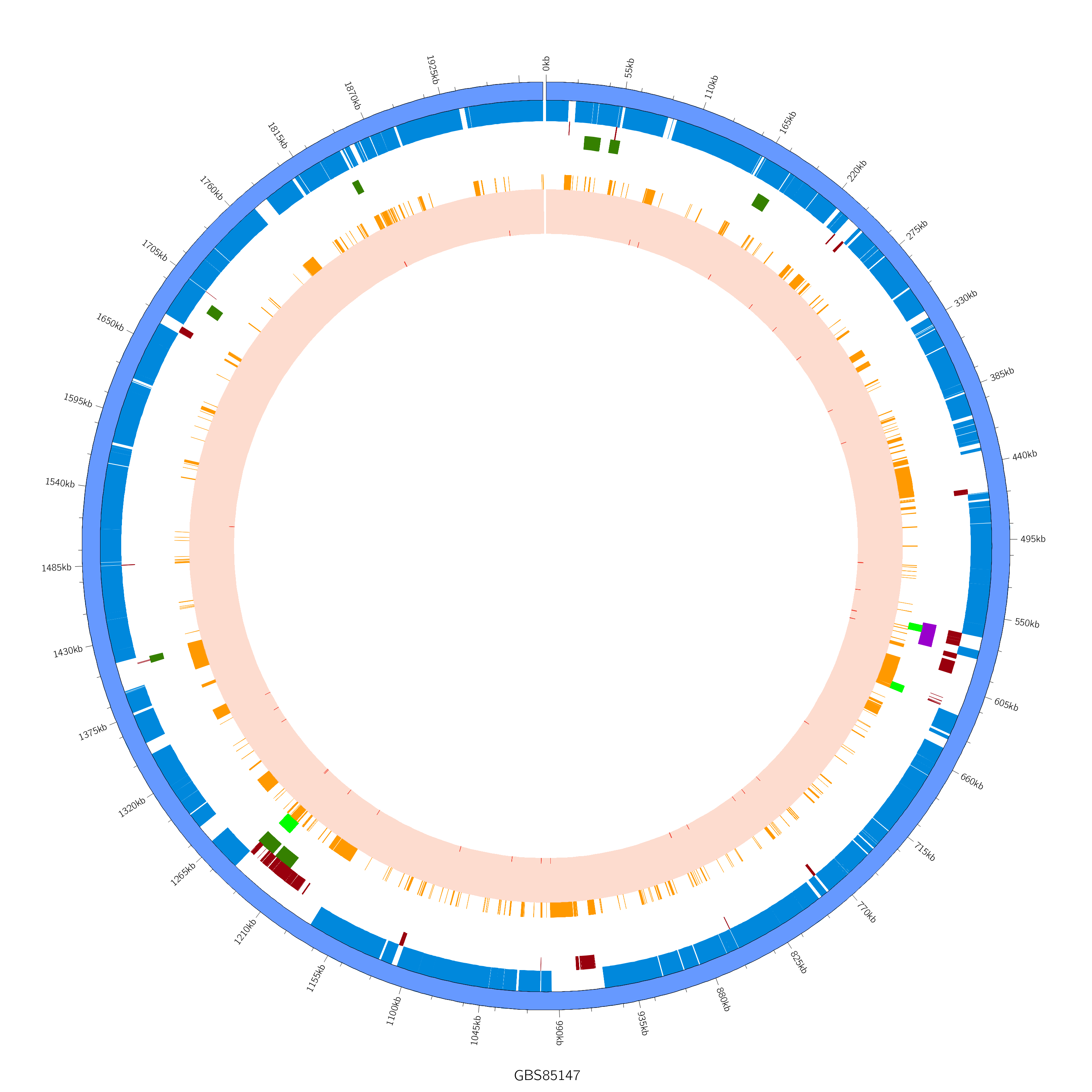

Supplement: Supplementary file 7 — Circos plots for all individual GBS genomes analyzed. All Additional file 4 information is plotted over genome extension. The tracks and the color code follow the same pattern as in Fig. 1. (ZIP 10957 kb) [file 12864_2018_4951_MOESM7_ESM.zip › Additional file 6/CP010319.png]

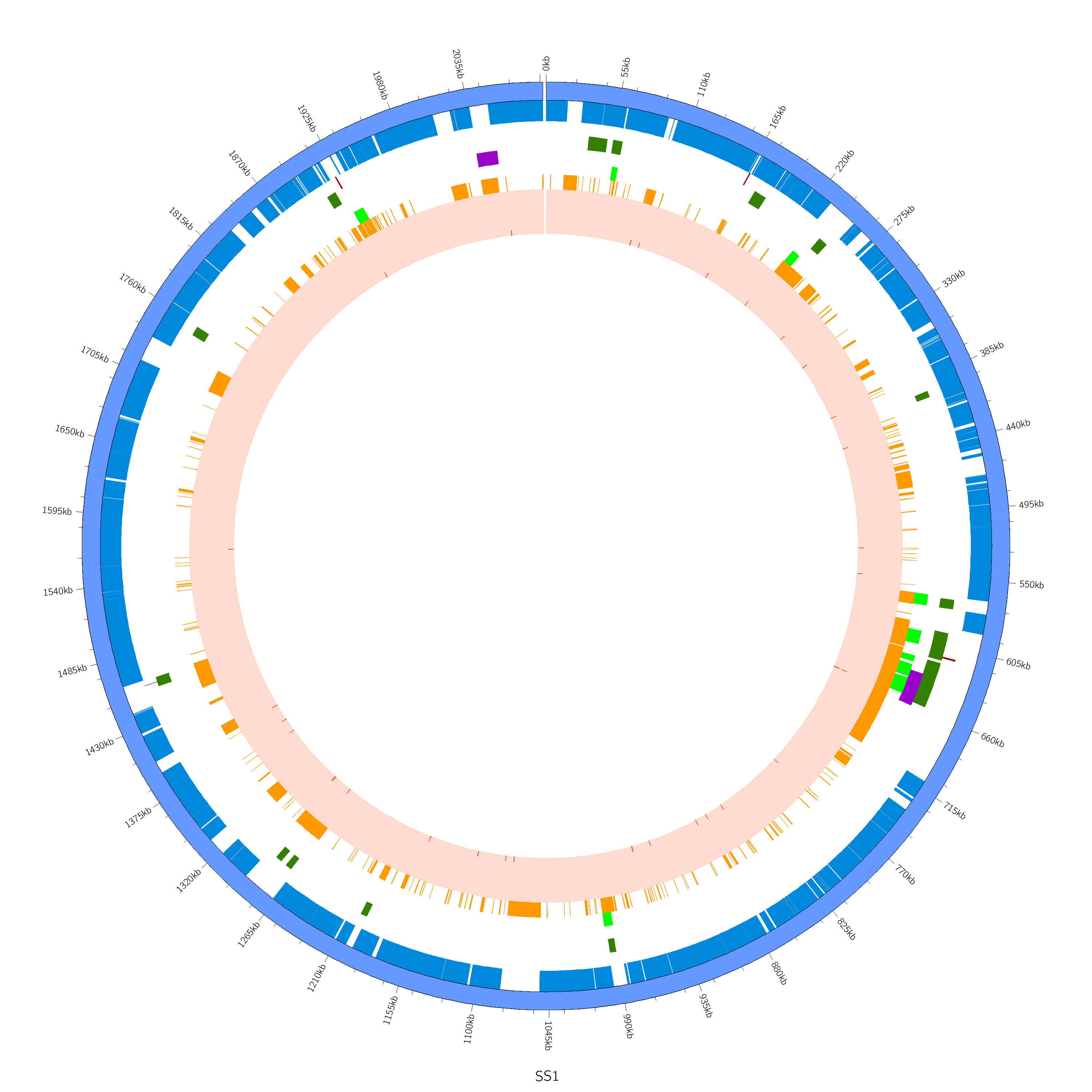

Supplement: Supplementary file 7 — Circos plots for all individual GBS genomes analyzed. All Additional file 4 information is plotted over genome extension. The tracks and the color code follow the same pattern as in Fig. 1. (ZIP 10957 kb) [file 12864_2018_4951_MOESM7_ESM.zip › Additional file 6/CP010867.png]

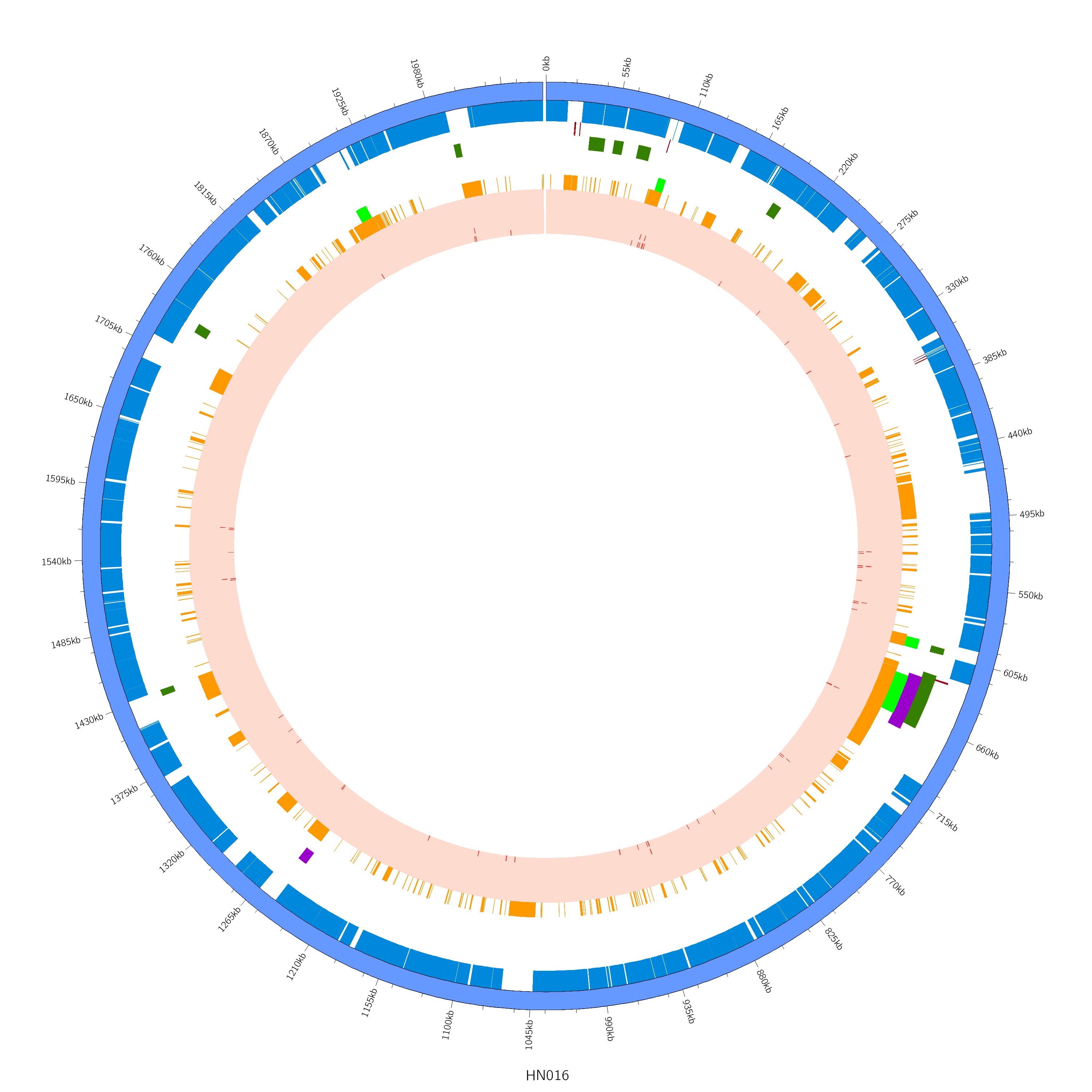

Supplement: Supplementary file 7 — Circos plots for all individual GBS genomes analyzed. All Additional file 4 information is plotted over genome extension. The tracks and the color code follow the same pattern as in Fig. 1. (ZIP 10957 kb) [file 12864_2018_4951_MOESM7_ESM.zip › Additional file 6/CP011325.png]

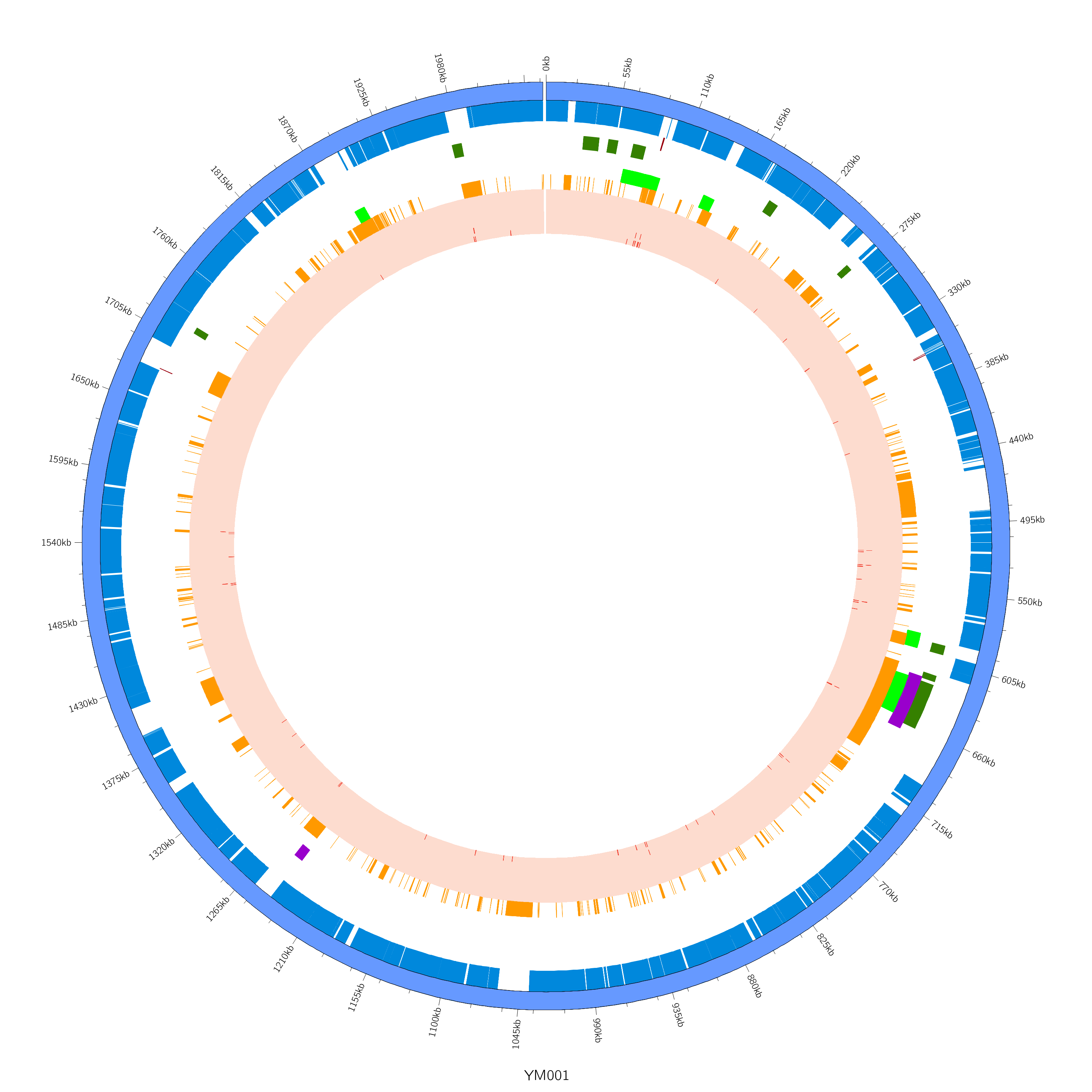

Supplement: Supplementary file 7 — Circos plots for all individual GBS genomes analyzed. All Additional file 4 information is plotted over genome extension. The tracks and the color code follow the same pattern as in Fig. 1. (ZIP 10957 kb) [file 12864_2018_4951_MOESM7_ESM.zip › Additional file 6/CP011326.png]

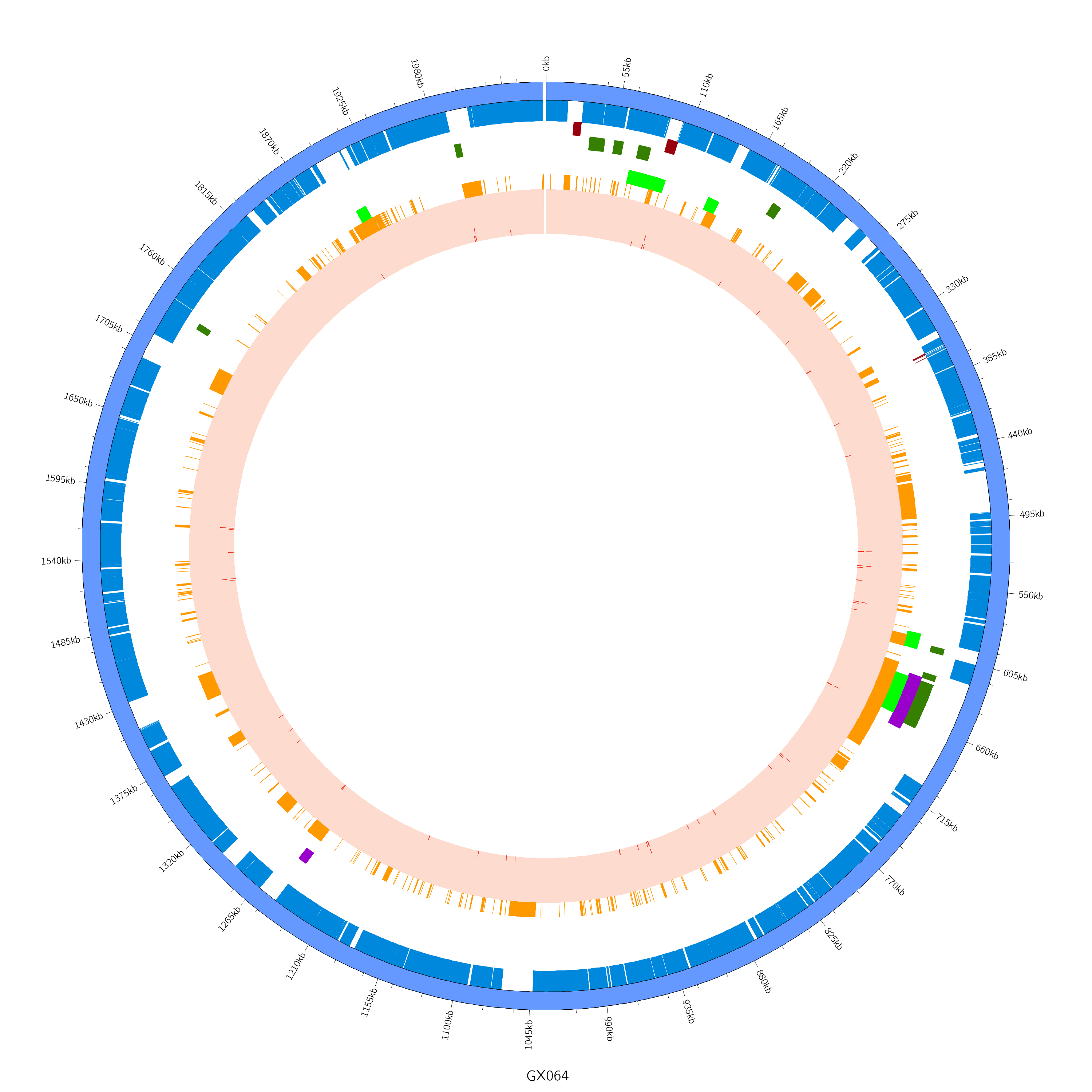

Supplement: Supplementary file 7 — Circos plots for all individual GBS genomes analyzed. All Additional file 4 information is plotted over genome extension. The tracks and the color code follow the same pattern as in Fig. 1. (ZIP 10957 kb) [file 12864_2018_4951_MOESM7_ESM.zip › Additional file 6/CP011327.png]

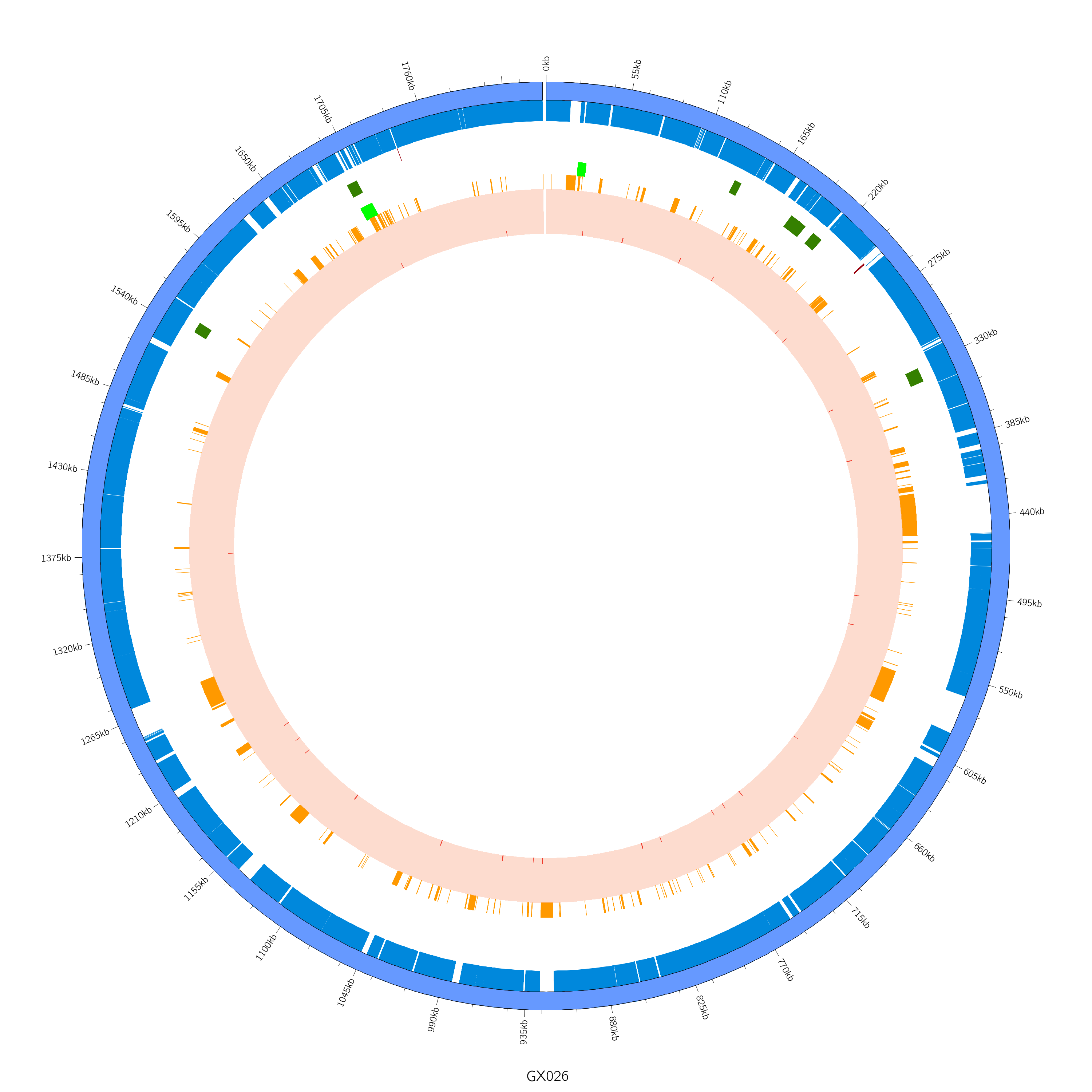

Supplement: Supplementary file 7 — Circos plots for all individual GBS genomes analyzed. All Additional file 4 information is plotted over genome extension. The tracks and the color code follow the same pattern as in Fig. 1. (ZIP 10957 kb) [file 12864_2018_4951_MOESM7_ESM.zip › Additional file 6/CP011328.png]

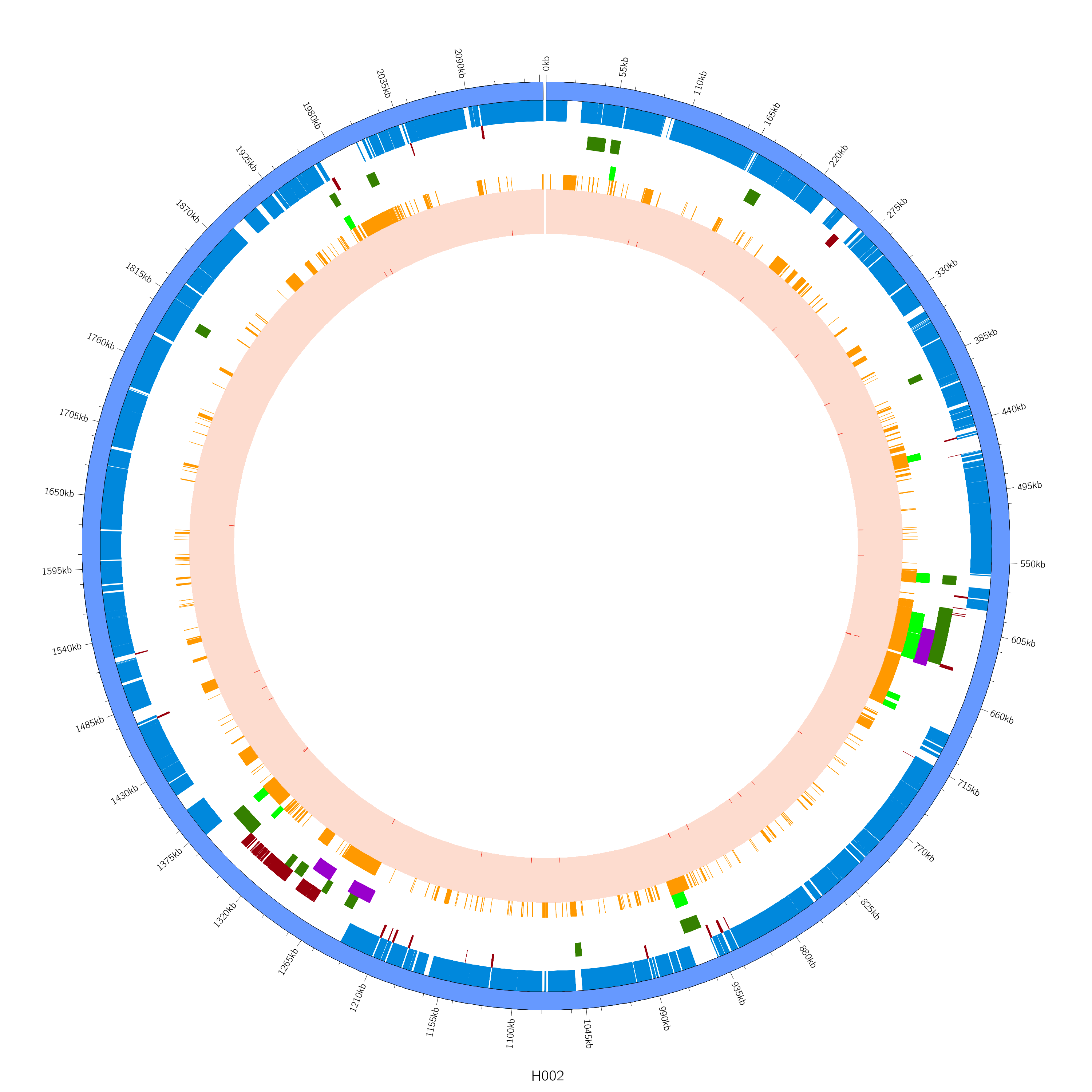

Supplement: Supplementary file 7 — Circos plots for all individual GBS genomes analyzed. All Additional file 4 information is plotted over genome extension. The tracks and the color code follow the same pattern as in Fig. 1. (ZIP 10957 kb) [file 12864_2018_4951_MOESM7_ESM.zip › Additional file 6/CP011329.png]

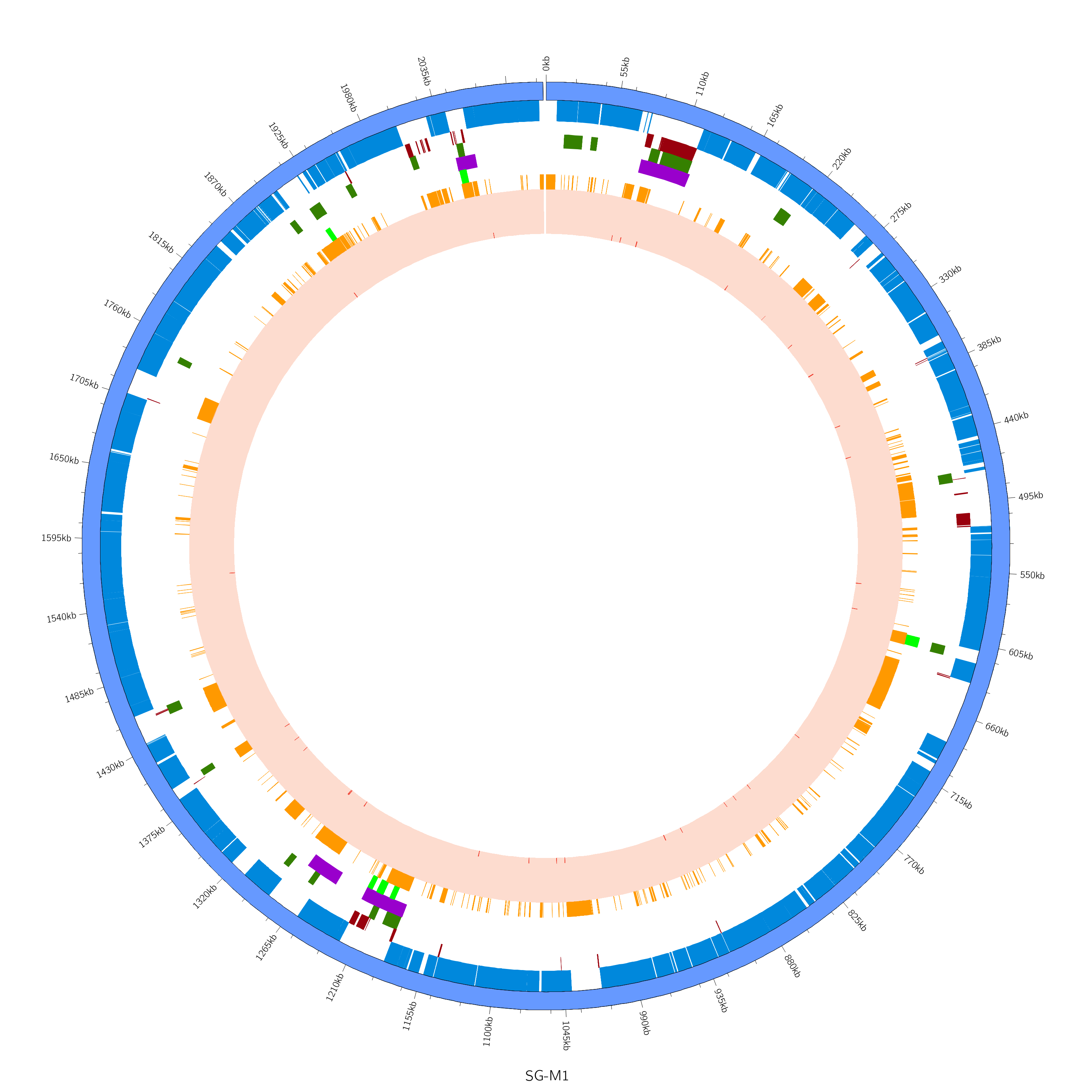

Supplement: Supplementary file 7 — Circos plots for all individual GBS genomes analyzed. All Additional file 4 information is plotted over genome extension. The tracks and the color code follow the same pattern as in Fig. 1. (ZIP 10957 kb) [file 12864_2018_4951_MOESM7_ESM.zip › Additional file 6/CP012419.png]

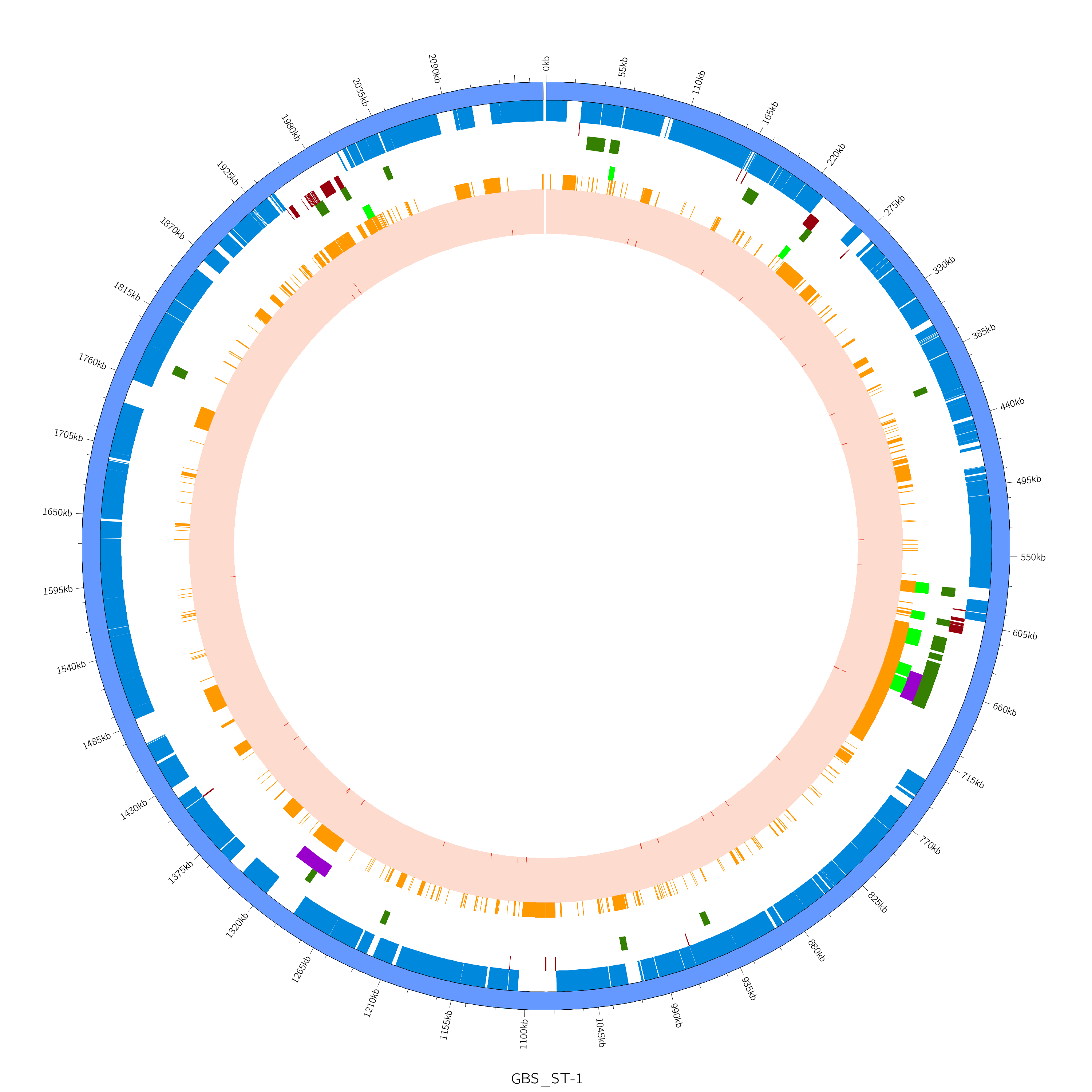

Supplement: Supplementary file 7 — Circos plots for all individual GBS genomes analyzed. All Additional file 4 information is plotted over genome extension. The tracks and the color code follow the same pattern as in Fig. 1. (ZIP 10957 kb) [file 12864_2018_4951_MOESM7_ESM.zip › Additional file 6/CP013202.png]

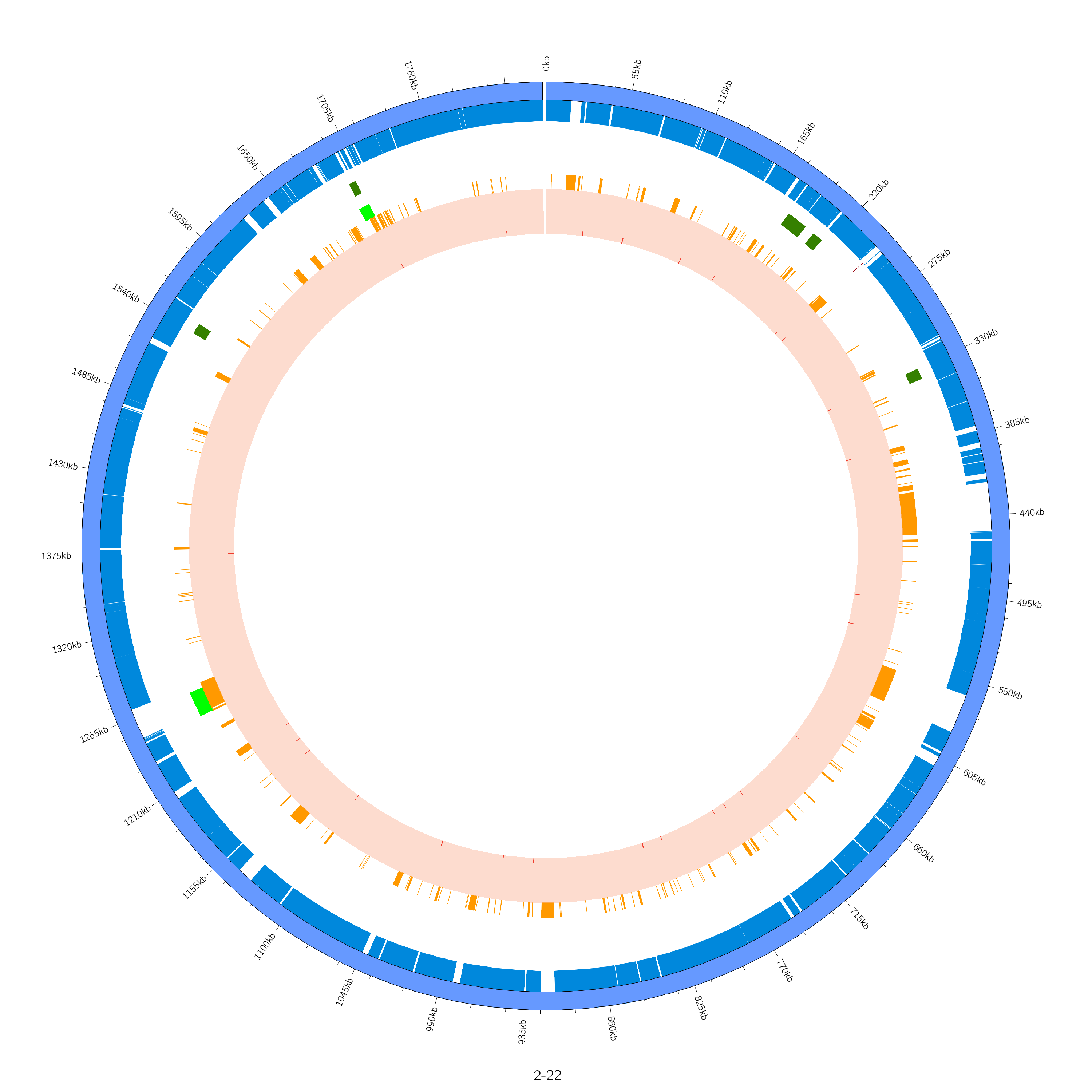

Supplement: Supplementary file 7 — Circos plots for all individual GBS genomes analyzed. All Additional file 4 information is plotted over genome extension. The tracks and the color code follow the same pattern as in Fig. 1. (ZIP 10957 kb) [file 12864_2018_4951_MOESM7_ESM.zip › Additional file 6/FO393392.png]

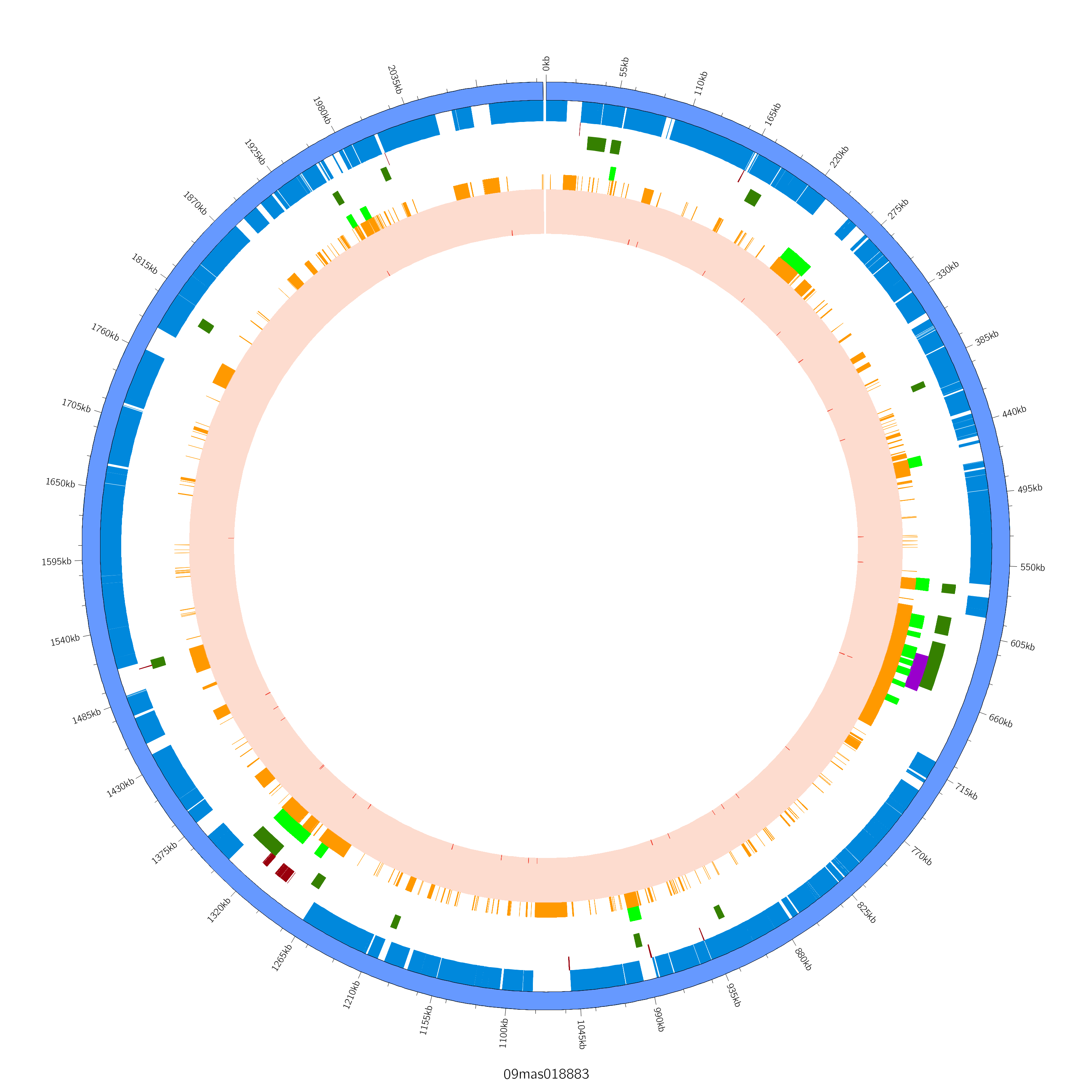

Supplement: Supplementary file 7 — Circos plots for all individual GBS genomes analyzed. All Additional file 4 information is plotted over genome extension. The tracks and the color code follow the same pattern as in Fig. 1. (ZIP 10957 kb) [file 12864_2018_4951_MOESM7_ESM.zip › Additional file 6/HF952104.png]

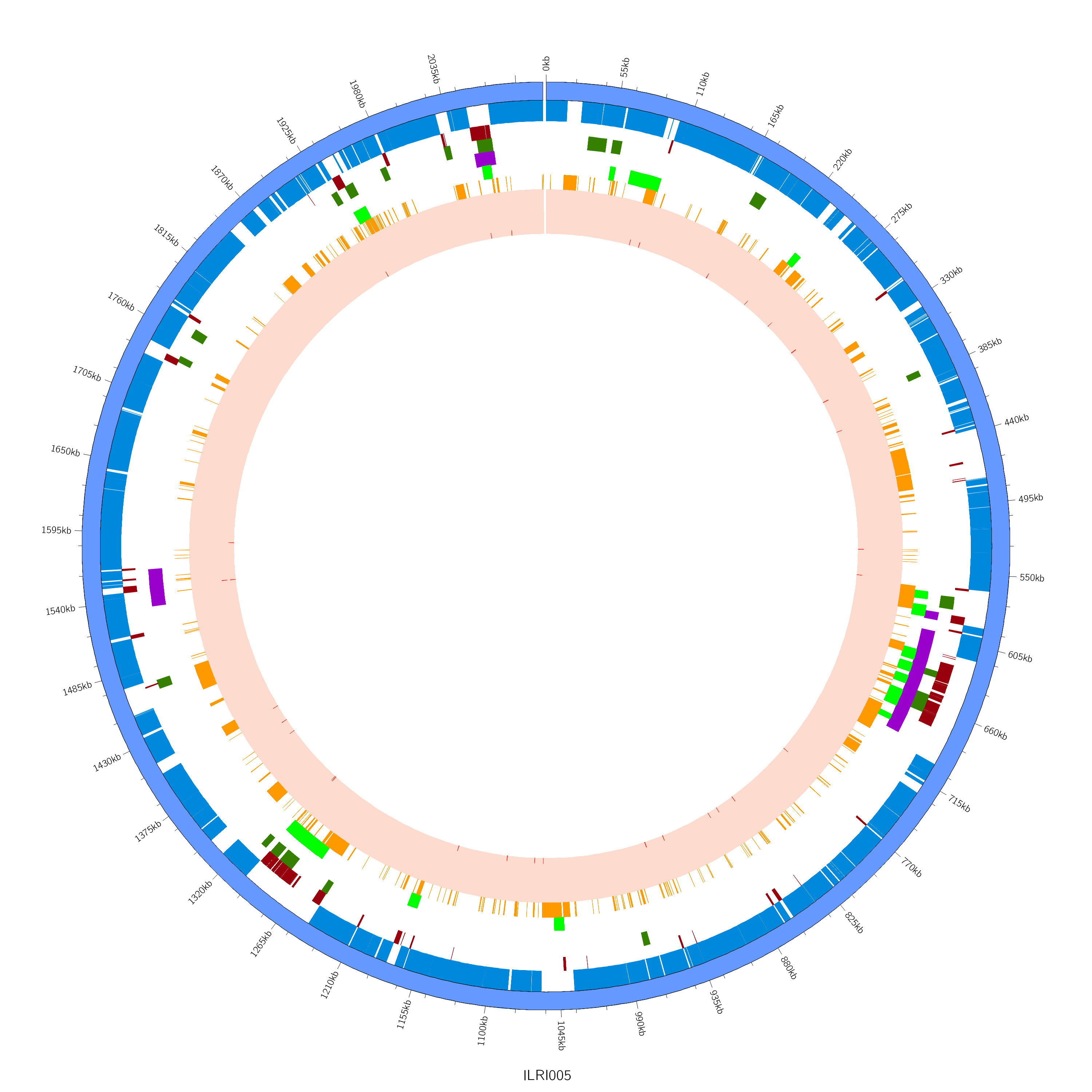

Supplement: Supplementary file 7 — Circos plots for all individual GBS genomes analyzed. All Additional file 4 information is plotted over genome extension. The tracks and the color code follow the same pattern as in Fig. 1. (ZIP 10957 kb) [file 12864_2018_4951_MOESM7_ESM.zip › Additional file 6/HF952105.png]

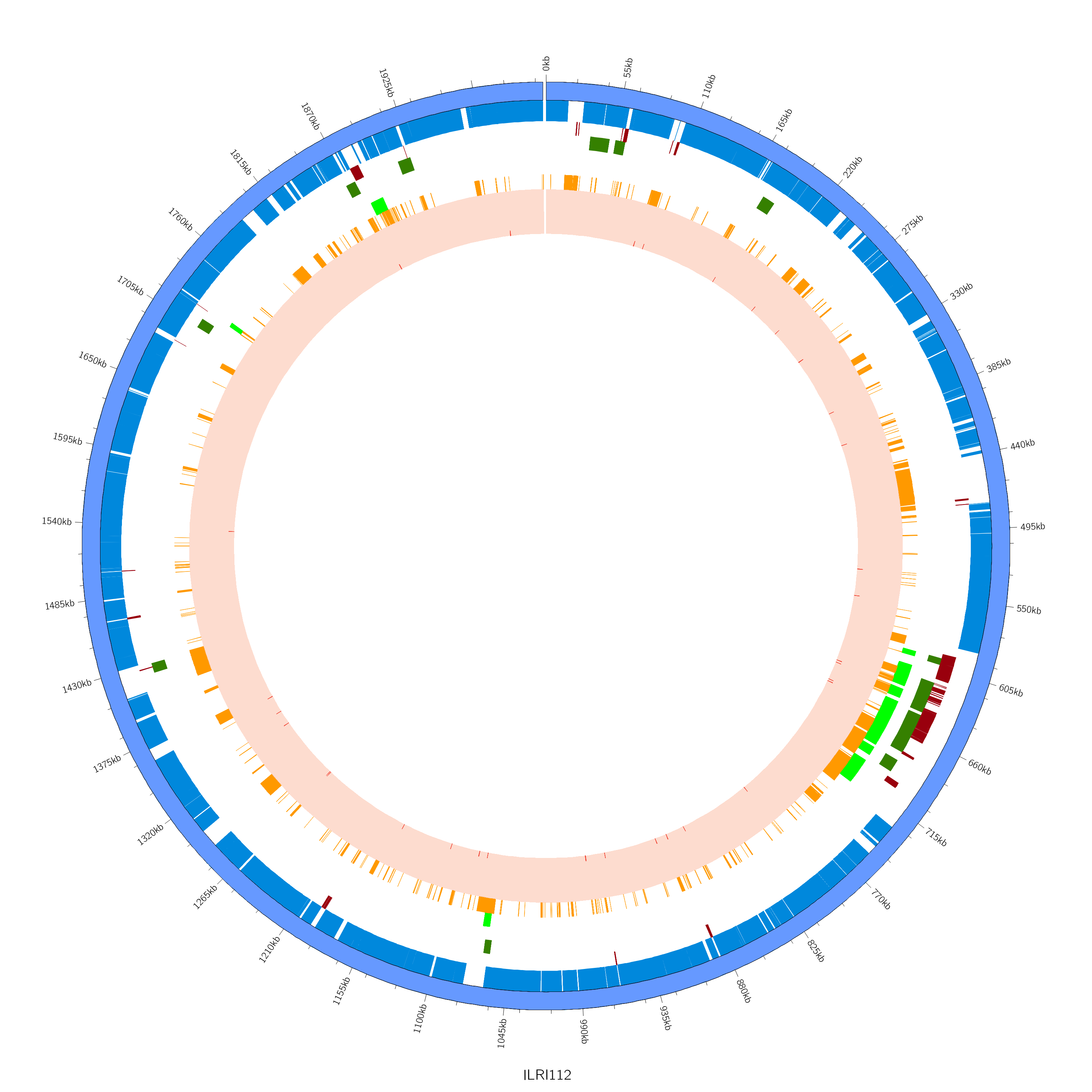

Supplement: Supplementary file 7 — Circos plots for all individual GBS genomes analyzed. All Additional file 4 information is plotted over genome extension. The tracks and the color code follow the same pattern as in Fig. 1. (ZIP 10957 kb) [file 12864_2018_4951_MOESM7_ESM.zip › Additional file 6/HF952106.png]

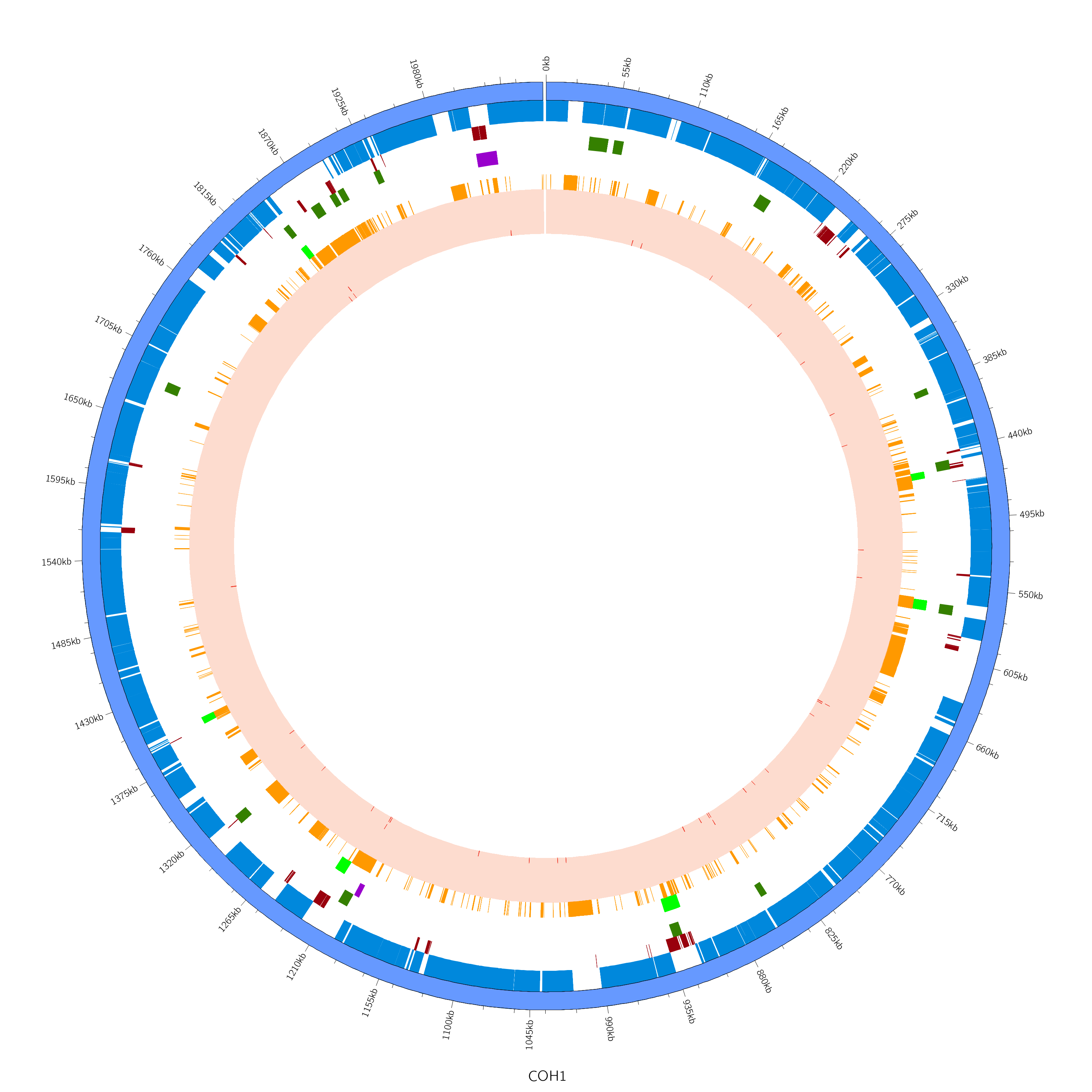

Supplement: Supplementary file 7 — Circos plots for all individual GBS genomes analyzed. All Additional file 4 information is plotted over genome extension. The tracks and the color code follow the same pattern as in Fig. 1. (ZIP 10957 kb) [file 12864_2018_4951_MOESM7_ESM.zip › Additional file 6/HG939456.png]

rl38

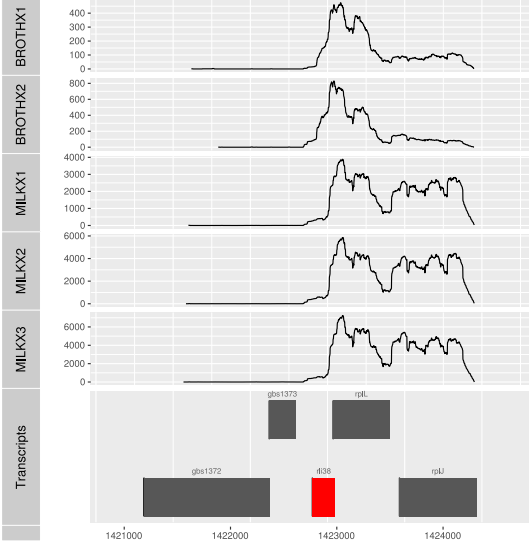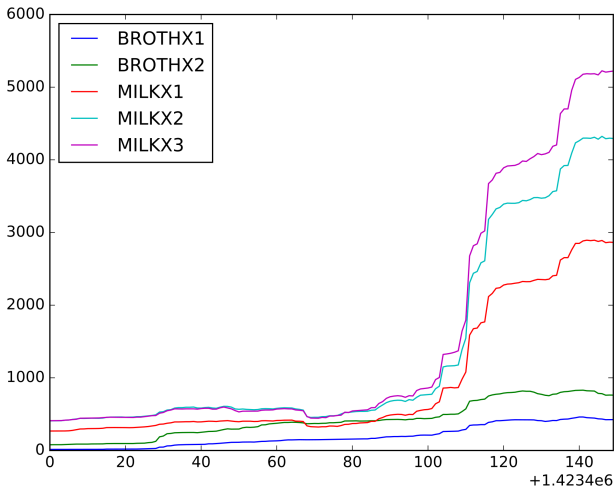

sau-50

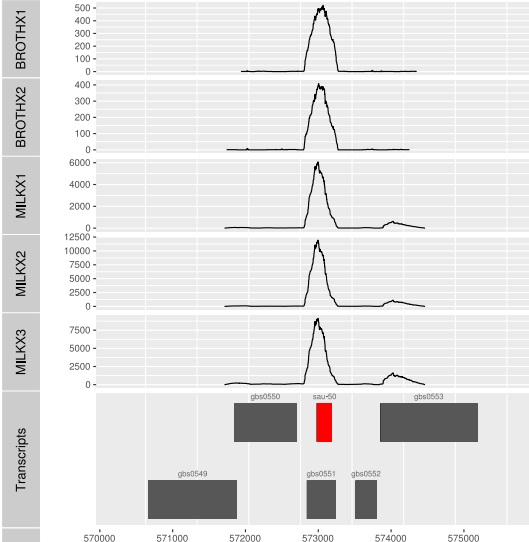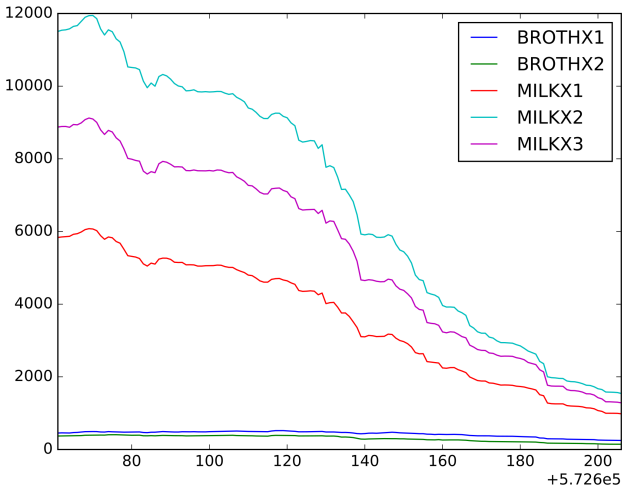

SSRC34

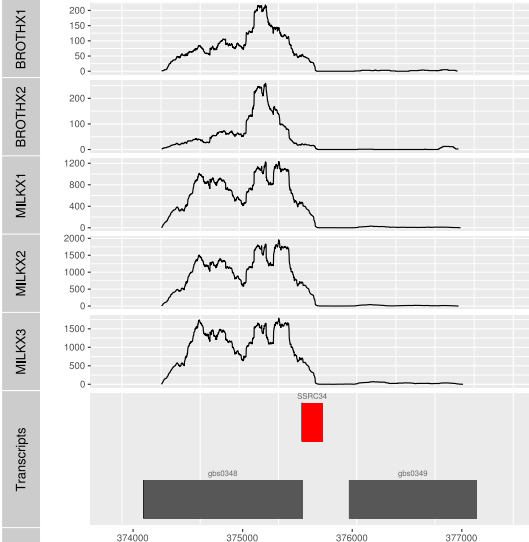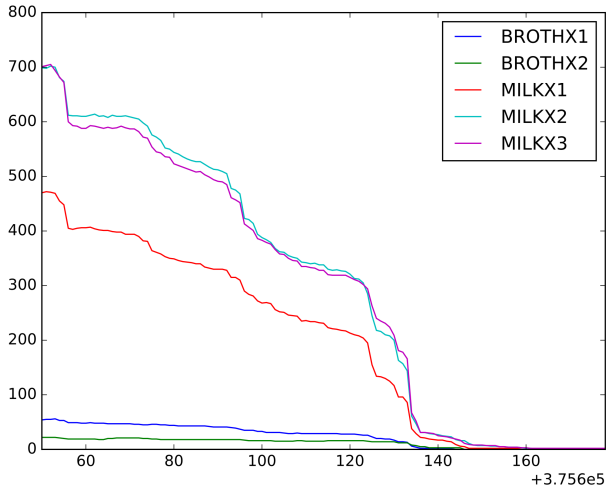

SSRC38

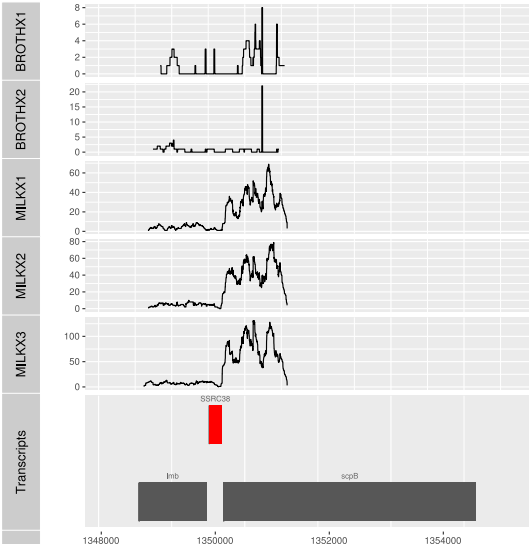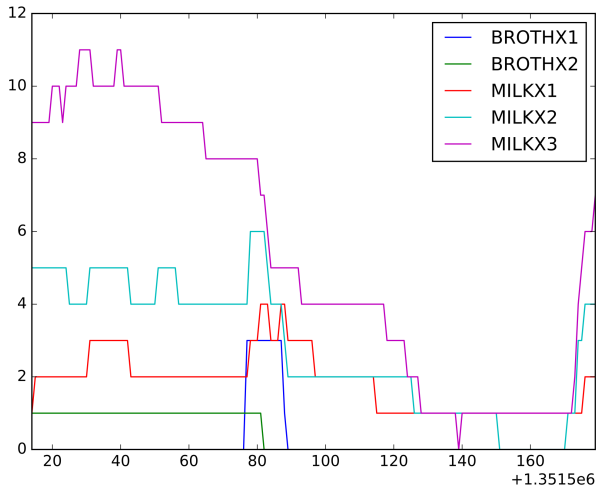

Supplement: Supplementary file 8 — Coverage plots for surrounding region of new detected RNA families in strain NEM316. (PDF 1039 kb) [file 12864_2018_4951_MOESM8_ESM.pdf]
