# Supplementary material for: Functional annotation and distribution overview of RNA families in 27 Streptococcus agalactiae genomes
Source: BMC Genomics. 2018 Jul 28;19:556. doi: 10.1186/s12864-018-4951-z (PMC6064168; doi:10.1186/s12864-018-4951-z)
Supplement: Supplementary file 11 — rli and RatA RNA families counts and phage association information. (DOCX 59 kb) [file 12864_2018_4951_MOESM11_ESM.docx]

| **Genome** | **Strain** | **rliA** | **RatA** | **phage** | **adjacent** |
| --- | --- | --- | --- | --- | --- |
| CP003810 | GD201008-001 | 1 | 2 | + | + |
| CP007572 | GBS6 |  |  |  |  |
| CP007571 | GBS2-NM |  |  |  |  |
| CP007570 | GBS1-NY |  |  |  |  |
| CP011325 | HN016 |  |  |  |  |
| CP011326 | YM001 | 1 | 2 | + | + |
| CP011327 | GX064 | 1 | 2 | + | + |
| AE009948 | 2603V/R | 1 | 1 |  |  |
| CP000114 | A909 | 1 | 1 | + | + |
| AL732656 | NEM316 |  |  |  |  |
| HF952104 | 09mas018883 | 1 | 1 | + | + |
| HF952106 | ILRI112 | 1 | 1 | + | + |
| HF952105 | ILRI005 | 1 | 1 | + | + |
| HG939456 | COH1 | 1 | 1 | + | + |
| CP007631 | NGBS061 |  |  |  |  |
| CP007632 | NGBS572 | 1 | 2 | + | + |
| CP006910 | CNCTC_10/84 |  |  |  |  |
| CP010867 | SS1 | 2 | 2 | + | + |
| CP011329 | H002 | 1 | 2 | + | + |
| CP010319 | GBS85147 | 1 | 1 | + | + |
| CP012419 | SG-M1 | 2 | 2 | + | + |
| CP013202 | GBS_ST-1 |  |  |  |  |
| CP003919 | SA20-06 | 1 | 2 | + | + |
| CP007482 | 138P |  |  |  |  |
| CP007565 | 138spar |  |  |  |  |
| FO393392 | 2-22 |  |  |  |  |
| CP011328 | GX026 |  |  |  |  |
