# Supplementary material for: Functional annotation and distribution overview of RNA families in 27 Streptococcus agalactiae genomes
Source: BMC Genomics. 2018 Jul 28;19:556. doi: 10.1186/s12864-018-4951-z (PMC6064168; doi:10.1186/s12864-018-4951-z)
Supplement: Supplementary file 6 — Plots of Features vs Cluster Assignments obtained on Weka by EM algorithm. (PDF 16 kb) [file 12864_2018_4951_MOESM6_ESM.pdf]

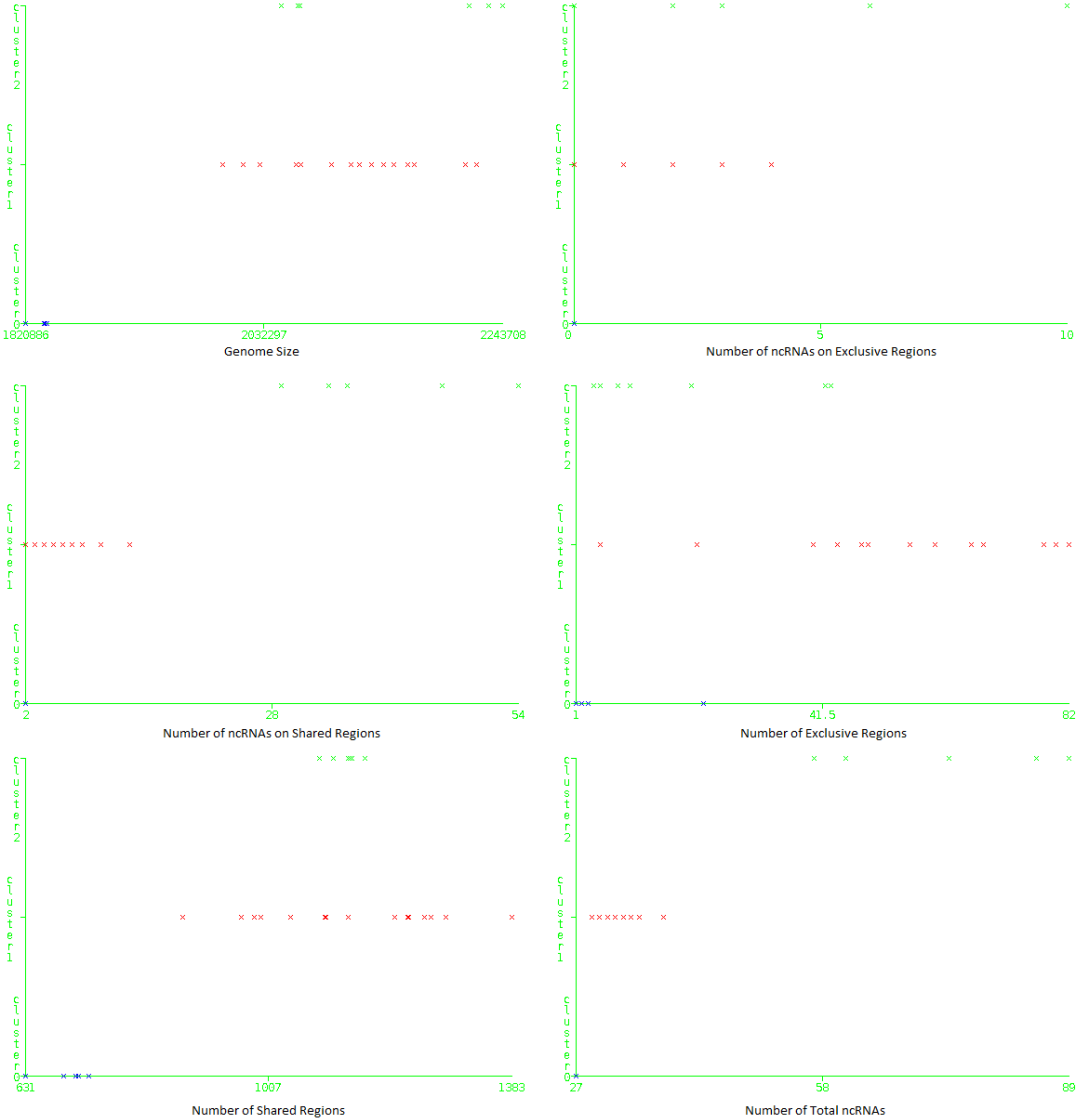
cluster2
cluster1
cluster0
1820886
2032297
2243708
Genome Size
0
5
10
Number of ncRNAs on Exclusive Regions
2
28
54
Number of ncRNAs on Shared Regions
1
41.5
82
Number of Exclusive Regions
631
1007
1383
Number of Shared Regions
27
58
89
Number of Total ncRNAs
